# Waist circumference partially mediates the non-linear association between TyHGB and MASLD in non-diabetic Japanese adults

**DOI:** 10.3389/fnut.2026.1915937

**Published:** 2026-07-24

**Authors:** Yanyan Xuan, Jingbo Yu, Qi Yao

**Affiliations:** 1 Department of Hospital Infection, The First Affiliated Hospital of Ningbo University, Ningbo, Zhejiang, China; 2 Department of Hepatology, The First Affiliated Hospital of Ningbo University, Ningbo, Zhejiang, China; 3 Department of Geriatrics Medicine, The First Affiliated Hospital of Ningbo University, Ningbo, Zhejiang, China

**Keywords:** metabolic dysfunction-associated steatotic liver disease, non-linear association, nutritional biomarker, TyHGB, visceral adiposity, waist circumference

## Abstract

**Background:**

The triglyceride high-density cholesterol-glucose body index (TyHGB) integrates atherogenic dyslipidemia, glucose metabolism, and generalized obesity, aligning closely with the pathophysiology of metabolic dysfunction-associated steatotic liver disease (MASLD). However, whether its association with MASLD follows a non-linear pattern and to what extent waist circumference (WC)-a surrogate for visceral adiposity-mediates this relationship remains unclear.

**Methods:**

This cross-sectional study included 13,682 non-diabetic Japanese adults from a community-based cohort. Multivariable logistic regression, generalized additive models, and two-piecewise linear regression were used to evaluate the independent and non-linear association between TyHGB and prevalent MASLD. Mediation analysis with 1,000 bootstrap resamples quantified the mediating effect of WC and its heterogeneity across metabolic subgroups.

**Results:**

The overall prevalence of MASLD was 15.25%. Each 1-unit increase in TyHGB was associated with a 1.35-fold higher odds of MASLD (OR = 1.35, 95% CI: 1.31–1.40, *P* < 0.001). A non-linear inflection point was identified at TyHGB = 9.70; below this threshold, the association was markedly stronger (OR=2.02, 95% CI: 1.88–2.17), whereas above it the risk increment plateaued (OR = 1.04, *P* = 0.090). WC partially mediated 46.4% of the TyHGB–MASLD association, with a substantially larger mediated proportion in non-obese individuals (42.67%) than in obese participants (7.83%).

**Conclusion:**

In non-diabetic Japanese adults, TyHGB is independently and non-linearly associated with MASLD, with WC partially mediating this relationship. The marked attenuation of WC mediation among obese individuals suggests a relative shift towards non-WC-mediated pathways under high metabolic burden. TyHGB may serve as a practical composite surrogate marker for MASLD risk stratification in community settings, particularly for non-obese individuals in whom WC captures a larger share of the risk pathway and who are frequently missed by BMI-based assessment.

## Introduction

Metabolic dysfunction-associated steatotic liver disease (MASLD) has superseded non-alcoholic fatty liver disease (NAFLD) as the predominant chronic liver disease globally, with an estimated one-third of the global adult population affected (1–7). An international expert consensus redefined this condition as MASLD in 2020, moving away from the traditional exclusionary diagnostic framework toward a positive criterion focused on metabolic dysfunction. To our knowledge, this paradigm shift likely better captures the disease’s underlying pathophysiology (8). Beyond its well-documented association with increased risks of liver cirrhosis and hepatocellular carcinoma, MASLD is also closely linked to several extrahepatic comorbidities—including cardiovascular diseases and type 2 diabetes (T2DM)—making it a global public health concern with an urgent unmet need for targeted interventions (9–13).

Early risk stratification is critical for reducing the disease burden of MASLD and is a core component of its prevention and control strategies. Liver biopsy is widely recognized as the gold standard for histopathological diagnosis of liver diseases, yet its invasive nature restricts its utility in large-scale population screening (14–16). Even non-invasive alternatives, such as abdominal ultrasound, have critical limitations for population-based screening. While a routine clinical tool, it lacks sufficient sensitivity for detecting mild hepatic steatosis. Its diagnostic accuracy also varies considerably between operators, which may limit precise population-based risk stratification (17, 18). These limitations highlight the urgent need for simple, non-invasive biomarkers—preferably derived from routine clinical parameters—to support early screening and risk stratification in community and nutritional epidemiology settings (19).

Since insulin resistance (IR) and visceral adiposity are central to MASLD pathogenesis, biomarkers reflecting these pathways are particularly attractive. IR and chronic low-grade inflammation constitute the core pathophysiological basis of MASLD, with abnormal visceral fat accumulation acting as a pivotal hub in disease development (20–23). Accumulating evidence confirms that waist circumference (WC)—a simple, readily available surrogate marker for visceral adiposity—outperforms body mass index (BMI) in assessing both the risk of MASLD onset and progression (24, 25). Central obesity-mediated metabolic dysfunction and lipotoxicity have been identified as critical independent risk factors for MASLD (26). This finding underscores the notable limitations of relying solely on BMI to capture obesity-related metabolic risk in the context of MASLD.

Against this background, metabolic indices that integrate lipid, glucose, and adiposity profiles have emerged as promising screening tools. The triglyceride-glucose (TyG) index is widely utilized as a surrogate marker of IR, and several studies have validated its close association with the onset of MASLD and its related complications (27, 28). Yet the TyG index primarily reflects fasting gluco-lipid dynamics. It does not explicitly account for protective lipid profiles (HDL-C) or the degree of generalized obesity—factors integral to MASLD pathogenesis. As such, it may inadequately reflect systemic metabolic dysregulation in this patient population. To address these inherent constraints, a novel composite metric, the triglyceride high-density cholesterol-glucose body index (TyHGB), was recently proposed (29). By integrating the TG-to-HDL-C ratio, FBG, and BMI, the TyHGB index captures synergistic effects of atherogenic dyslipidemia, glucose metabolic disorders, and generalized obesity. Emerging studies show promising potential for TyHGB in risk assessment of metabolism-related diseases, laying a theoretical foundation for its use in MASLD screening (30–33).

Despite these advances, critical gaps remain. To date, most evidence derives from the earlier NAFLD framework and often includes patients with diabetes (34), whose metabolic perturbations may confound the true association between TyHGB and early metabolic steatosis. A critical pathophysiological question persists regarding the extent to which visceral adiposity—proximally measured by WC—mediates the association between TyHGB and MASLD risk. Visceral adiposity promotes excessive lipid accumulation, adipose inflammation, and dysregulated adipokine release—key drivers of hepatic lipotoxicity and metabolic dysfunction—providing a strong biological rationale for WC as a mediator. Elucidating this pathway is critical to understanding TyHGB’s mechanism in MASLD pathogenesis.

To address these critical gaps, we conducted a cross-sectional analysis among 13,682 non-diabetic Japanese adults. We aimed to: (1) characterize the independent and non-linear association between TyHGB and prevalent MASLD; (2) quantify the mediating role of WC—a surrogate for visceral adiposity—in this association and its heterogeneity across metabolic subgroups; and (3) compare the discriminative performance of TyHGB against conventional metabolic markers. It was hypothesized that findings would support TyHGB as a simple, non-invasive tool for early MASLD risk stratification in community settings.

## Materials and methods

### Study design and population

This study was a cross-sectional secondary analysis of anonymized data from the Non-Alcoholic Fatty Liver Disease in the Gifu Area Longitudinal Analysis (NAGALA) cohort. This population-based prospective health screening program was initiated in 1994 at Murakami Memorial Hospital in Gifu Prefecture, Japan. The primary objective of the NAGALA study is to clarify the epidemiological characteristics and metabolic risk factors associated with MASLD and T2DM, to support clinical and public health decision-making.

Participants were individuals who received regular health check-ups at the hospital. Approximately 60% of participants attended repeated examinations at least once per year, and more than 8,000 health screening records were accumulated annually, creating a high-quality longitudinal dataset with minimal loss to follow-up. All data collection, including demographic factors, physical measurements, biochemical tests, lifestyle information, and past medical history, was conducted by trained medical professionals under standardized operating procedures to ensure data consistency and validity.

Professor Takuro Okamura shared the de-identified dataset, which is publicly accessible through the Dryad Digital Repository.^1^ This study fully complied with the repository’s data-use policies. Ethical approval for the original NAGALA study was obtained from the Ethics Committee of Murakami Memorial Hospital. All participants provided written informed consent for the use of their anonymized data in future research. Since this study involved only publicly available, anonymized data, no additional ethical approval was required under the Declaration of Helsinki. All analytical procedures were conducted in accordance with international ethical guidelines.

**FIGURE 1:**
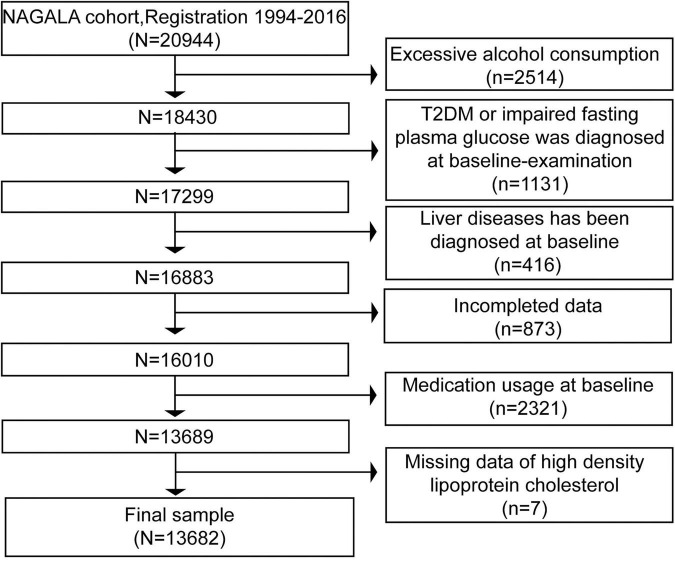
Flowchart of the study participants.the NAGALA, Non-Alcoholic Fatty Liver Disease in the Gifu Area Longitudinal Analysis; T2DM, Type 2 diabetes.

We initially enrolled 20,944 Japanese adults who underwent health examinations from 1994 to 2016. To minimize bias and ensure a homogeneous study sample, we excluded participants with: (1) heavy alcohol consumption (*n* = 2,514); (2) diabetes or impaired fasting glucose (*n* = 1,131);(3) any known chronic liver disease (*n* = 416); (4) incomplete essential baseline data (*n* = 873); (5) regular medication use (*n* = 2,321); (6) missing HDL-C values (*n* = 7). After exclusion of 7,262 participants, 13,682 participants were included in the final analysis. The detailed participant enrollment process is presented in Figure 1.

### Definition of MASLD

MASLD was defined in line with the 2020 international expert consensus (8). Diagnosis of MASLD required evidence of hepatic steatosis together with at least one indicator of metabolic dysfunction.

Hepatic steatosis was assessed by abdominal ultrasonography conducted by qualified sonographers, using standardized protocols across all subjects. Steatosis was diagnosed based on four characteristic imaging findings: lighter hepatic parenchyma relative to the renal cortex, obscured borders of intrahepatic vessels, increased hepatorenal echogenic difference, and distal acoustic attenuation.

For the diagnosis of MASLD, participants were required to fulfill one of the following three metabolic criteria: (1) Overweight or obesity (BMI ≥ 23 kg/m^2^, adapted for Asian populations); (2) Established T2DM;(3) Two or more metabolic risk abnormalities, including: Abdominal obesity (WC ≥ 90 cm in men or ≥ 80 cm in women); Hypertension (blood pressure ≥ 130/85 mmHg or use of antihypertensive drugs); Hypertriglyceridemia (TG ≥ 1.7 mmol/L or lipid-lowering medication); Low HDL-C (<1.0 mmol/L in men or < 1.3 mmol/L in women or lipid-lowering treatment); Prediabetes (fasting blood glucose 5.6–6.9 mmol/L or Glycated hemoglobin 5.7–6.4%).

### Definition of TyHGB

The TyHGB index is a recently developed composite index that refines the TyG index by incorporating HDL-C and BMI to better assess IR and metabolic risk. It was calculated using the following formula: TyHGB = TG (mmol/L)/HDL-C (mmol/L) + 0.7 × FBG (mmol/L) + 0.1 × BMI (kg/m^2^).

### Variables

Data for the present study were retrieved from the NAGALA database, with rigorous adherence to standardized data extraction protocols designed to ensure the consistency and reliability of all measured variables. Trained researchers distributed a validated, structured questionnaire to collect participants’ demographic information (age, gender), anthropometric measurements (height, weight, WC), and lifestyle habits (smoking status, alcohol intake, exercise status, and intensity). Anthropometric assessments were performed by well-trained professional technicians using calibrated standardized instruments, in strict compliance with the standard operating procedures (SOPs) of the original cohort study. Lifestyle-related data were collected through standardized face-to-face interviews, consistent with the NAGALA cohort study’s methodological guidelines.

All participants provided venous blood samples after an overnight fast of at least 8 h. All biochemical detections were performed on a discrete-type automated biochemical analyzer, with strict quality control measures including daily calibration and internal quality control samples to ensure test result accuracy. The detected biochemical indicators encompassed serum lipid profiles [total cholesterol (TC), HDL-C, low-density lipoprotein cholesterol (LDL-C), triglycerides (TG)], glucose metabolism markers [fasting blood glucose (FBG), glycated hemoglobin (HbA1c)], and liver function enzymes [aspartate aminotransferase (AST), alanine aminotransferase (ALT), γ-glutamyltransferase (GGT)].

Based on participants’ self-reported smoking history, smoking status was categorized into three mutually exclusive groups: never smoked, former smokers (who had quit smoking for more than 6 months), and current smokers. exercise status levels were classified into two groups: regular exercisers (individuals engaging in moderate-to-vigorous exercise for at least 1 session per week, each lasting at least 30 min) and non-exercisers. Hypertension was defined in accordance with the 2019 Guidelines for the Management of Hypertension issued by the Japanese Society of Hypertension (JSH), which specifies that office systolic blood pressure (SBP) ≥ 140 mmHg and/or diastolic blood pressure (DBP) ≥ 90 mmHg, or regular use of antihypertensive medications at the time of enrollment (35). BMI was computed as weight in kilograms divided by the square of height in meters (kg/m^2^), a widely used indicator of body adiposity.

The primary outcome variable of this study was MASLD. The primary exposure variable was the TyHGB index, and the mediator variable was the WC. Covariates were selected based on previously published clinical studies and epidemiological evidence that have been confirmed to be associated with MASLD risk. Continuous covariates included age, BMI, LDL-C, AST, ALT, GGT, and HbA1c, while categorical covariates consisted of gender, smoking status, alcohol consumption status, exercise status, and hypertension status.

### Statistical analysis

All statistical analyses were performed using R software (version 4.1.0)^2^ and EmpowerStats (version 4.0).^3^ Baseline characteristics of participants were summarized according to quartiles of the TyHGB index. Normally distributed continuous variables are presented as mean ± standard deviation, whereas skewed variables are shown as median (interquartile range). Categorical variables are reported as counts and percentages.

Logistic regression models were applied to evaluate the association between the TyHGB index and MASLD. Three hierarchical models were established: a crude, unadjusted model; a model adjusted for age and sex; and a fully adjusted model further adjusted for hypertension, smoking status, alcohol consumption, exercise status, HbA1c, BMI, ALT, AST, GGT, and LDL-C.

Sensitivity analyses were conducted to assess the robustness of the main results. Stratified analyses were performed by sex, age group, BMI, WC, triglyceride category, and hypertension status. Generalized additive models (GAM) were used to explore potential non-linear relationships between TyHGB and MASLD risk. When a non-linear trend was observed on the smoothing curve, a two-piecewise linear regression model was applied to estimate the threshold effect.

Receiver operating characteristic (ROC) curve analysis was used to evaluate the diagnostic performance of TyHGB and other biochemical markers for MASLD. A two-sided *p*-value < 0.05 was considered statistically significant.

Mediation analysis was performed using the R package “mediation” to investigate whether WC mediated the association between TyHGB and MASLD. Linear regression was first used to examine the association between TyHGB and WC. Logistic regression was then applied to assess the relationship between WC and MASLD. The total effect of TyHGB on MASLD was separated into direct and indirect effects mediated by WC, and the proportion mediated was calculated as the ratio of the indirect effect to the total effect.

To mitigate potential mathematical coupling arising from the inclusion of BMI in the TyHGB formula and its high correlation with WC, we performed a supplementary mediation analysis. A modified index, TyHGB_noBMI, was constructed by excluding the BMI component: TyHGB_noBMI = TG (mmol/L)/HDL-C (mmol/L) + 0.7 × FBG (mmol/L). We then repeated the causal mediation analysis using TyHGB_noBMI as the exposure and WC as the mediator, while adjusting for the same set of covariates including BMI as a separate confounder.

Bootstrap resampling with 1,000 iterations was performed to obtain 95% confidence intervals (CIs). A mediation effect was considered statistically significant if the 95% CI did not include zero. A two-sided *p*-value < 0.05 was set as the level of statistical significance for all tests.

## Results

### Baseline characteristics of participants

This study included 13,682 subjects, of whom 2,087 were diagnosed with MASLD, corresponding to a prevalence of 15.25%. The mean age of the cohort was 43.38 ± 8.84 years, and 50.91% were male. TyHGB values were normally distributed, ranging from 3.90 to 44.03 with a mean (SD) of 7.43 (1.94). Detailed anthropometric and biochemical characteristics stratified by TyHGB quartiles are presented in Table 1.

**TABLE 1:** Baseline characteristics of the study population according to the TyHGB quartile group.

Characteristic	TyHGB quartile				*P*-value
	Q1(3.90-6.22)	Q2(6.22–6.93)	Q3(6.93-8.05)	Q4(8.05-44.03)	
No. of participants	3,421	3,420	3,420	3,421	
Age (years, mean ± SD)	40.41 ± 8.15	43.16 ± 8.67	44.92 ± 9.07	45.01 ± 8.67	<0.001
Age (years, N, %)					<0.001
<40	1721 (50.31%)	1296 (37.89%)	1083 (31.67%)	1057 (30.90%)	
≥ 40	1700 (49.69%)	2124 (62.11%)	2337 (68.33%)	2364 (69.10%)	
Sex (N, %)					<0.001
Male	2961 (86.55%)	2131 (62.31%)	1252 (36.61%)	622 (18.18%)	
Female	460 (13.45%)	1289 (37.69%)	2168 (63.39%)	2799 (81.82%)	
Smoking status (N, %)					<0.001
Never smoker	2841 (83.05%)	2453 (71.73%)	1873 (54.77%)	1483 (43.35%)	
Former smoker	308 (9.00%)	489 (14.30%)	727 (21.26%)	823 (24.06%)	
Current smoker	272 (7.95%)	478 (13.98%)	820 (23.98%)	1115 (32.59%)	
Drinking status (N, %)					<0.001
Never/light drinker	3114 (91.03%)	2965 (86.70%)	2855 (83.48%)	2868 (83.84%)	
Moderate drinker	252 (7.37%)	412 (12.05%)	548 (16.02%)	542 (15.84%)	
Heavy drinker	55 (1.61%)	43 (1.26%)	17 (0.50%)	11 (0.32%)	
Hypertension (N, %)	35 (1.02%)	92 (2.69%)	196 (5.73%)	413 (12.07%)	<0.001
Habit of exercise (N, %)	587 (17.16%)	611 (17.87%)	629 (18.39%)	519 (15.17%)	0.002
BMI (kg/m^2^, mean ± SD)	19.33 ± 1.73	21.09 ± 2.02	22.73 ± 2.43	24.90 ± 3.16	<0.001
BMI (kg/m^2^, N, %)					<0.001
<25	3415 (99.82%)	3315 (96.93%)	2865 (83.77%)	1938 (56.65%)	
≥ 25	6 (0.18%)	105 (3.07%)	555 (16.23%)	1483 (43.35%)	
Weight (kg, mean ± SD)	50.02 ± 6.28	56.31 ± 7.98	62.55 ± 9.04	70.61 ± 11.11	<0.001
Height (cm, mean ± SD)	160.71 ± 6.98	163.20 ± 8.46	165.71 ± 8.54	168.20 ± 7.95	<0.001
WC (cm, mean ± SD)	68.01 ± 5.62	73.01 ± 6.49	78.18 ± 6.88	84.45 ± 7.96	<0.001
SBP (mmHg, mean ± SD)	105.23 ± 12.05	110.89 ± 13.05	116.62 ± 13.85	121.65 ± 14.68	<0.001
DBP (mmHg, mean ± SD)	65.14 ± 8.53	68.67 ± 9.12	72.83 ± 9.62	76.77 ± 10.08	<0.001
ALT (IU/L, median, quartile)	13.00 (11.00-17.00)	16.00 (11.00-19.00)	18.00 (13.00-23.00)	23.00 (16.00-33.00)	<0.001
GGT (IU/L, median, quartile)	12.00 (10.00-14.00)	13.00 (10.00-16.25)	15.00 (12.00-21.00)	21.00 (15.00-31.00)	<0.001
AST (IU/L, median, quartile)	16.00 (13.00-19.00)	16.00 (13.00-20.00)	17.00 (14.0021.00)	19.00 (15.00-24.00)	<0.001
TG (mmol/L, median, quartile)	0.41 (0.30-0.51)	0.59 (0.47-0.72)	0.84 (0.68-0.99)	1.46 (1.15-1.89)	<0.001
TC (mmol/L, mean ± SD)	4.83 ± 0.79	5.00 ± 0.83	5.18 ± 0.85	5.47 ± 0.87	<0.001
HDL-C (mmol/L, mean ± SD)	1.81 ± 0.37	1.60 ± 0.33	1.37 ± 0.27	1.09 ± 0.22	<0.001
LDL-C (mmol/L, mean ± SD)	2.84 ± 0.66	3.12 ± 0.72	3.42 ± 0.75	3.63 ± 0.81	<0.001
FBG (mmol/L, mean ± SD)	4.78 ± 0.31	5.09 ± 0.33	5.27 ± 0.35	5.40 ± 0.36	<0.001
HbA1c (%, mean ± SD)	5.10 ± 0.29	5.16 ± 0.30	5.20 ± 0.32	5.26 ± 0.34	<0.001
MASLD (N, %)	4 (0.12%)	68 (1.99%)	437 (12.78%)	1578 (46.13%)	<0.001

Mean ± SD or medians (quartile interval) were for continuous variables. % was for categorical variables. MASLD, Metabolic dysfunction associated steatotic liver disease; TyHGB, Triglyceride high-density cholesterol-glucose body index; BMI, Body mass index; WC, Waist circumference; SBP, Systolic blood pressure; DBP, Diastolic blood pressure; ALT, Alanine aminotransferase; AST, Aspartate aminotransferase; GGT, Gamma-glutamyl transpeptidase; HbA1c, Glycosylated hemoglobin; FBG, Fasting blood glucose; TG, Triglycerides; TC, Total cholesterol; HDL-C, High-density lipoprotein cholesterol; LDL-C, Low-density lipoprotein cholesterol.

Multiple clinical and laboratory parameters were positively associated with increasing TyHGB levels, including BMI, blood pressure, glycemic indicators, lipid profiles, and liver enzymes (ALT, AST, GGT) (all *P* < 0.01). In comparison, HDL-C levels were inversely related to TyHGB. Higher TyHGB quartiles were accompanied by elevated proportions of participants aged ≥ 40 years, female, overweight or obese (BMI ≥ 25 kg/m^2^), smoking, MASLD, and hypertension. In contrast, lower TyHGB was associated with younger age, male, normal BMI, non-smoking status, and absence of hypertension or MASLD (all *P* < 0.01).

### Relationship between TyHGB and MASLD

Multivariate logistic regression was applied to examine the association between TyHGB and MASLD risk, with detailed results presented in Table 2. TyHGB was significantly and positively associated with MASLD risk in all three models (all *P* < 0.001). In unadjusted Model 1, each 1-unit increase in TyHGB was associated with an odds ratio (OR) of 2.07 (95% CI: 2.00–2.14). After adjustment for age and sex in Model 2, the association remained significant (OR = 1.91, 95% CI: 1.84–1.97). In fully adjusted Model 3 (adjusted for age, sex, hypertension, smoking, drinking, exercise, WC, HbA1c, ALT, AST, GGT, and LDL-C), the OR was 1.35 (95% CI: 1.31–1.40), confirming an independent positive link between TyHGB and MASLD risk.

**TABLE 2:** Associations between TyHGB and the MASLD risk.

Variable	Model 1 OR (95% CI), *P-*value	Model 2 OR (95% CI), *P-*value	Model 3 OR (95% CI), *P-*value
TyHGB	2.07 (2.00, 2.14) < 0.001	1.91 (1.84, 1.97) < 0.001	1.35 (1.31, 1.40) < 0.001
Q1 (3.90-6.22)	Reference	Reference	Reference
Q2 (6.22-6.93)	17.33 (6.31, 47.56) < 0.001	15.57 (5.67, 42.76) < 0.001	8.00 (2.71, 23.62) < 0.001
Q3 (6.93-8.05)	125.14 (46.70, 335.34) < 0.001	101.58 (37.79, 273.04) < 0.001	25.38 (8.75, 73.63) < 0.001
Q4 (8.05-44.03)	731.42 (273.73, 1954.39) < 0.001	556.01 (207.13, 1492.56) < 0.001	65.79 (22.64, 191.22) < 0.001
*P* for trend	<0.001	< 0.001	<0.001
Subgroup analysis			
Sex			
Men	2.86 (2.63, 3.11) <0.001	2.79 (2.56, 3.04) <0.001	1.73 (1.57, 1.90) <0.001
Women	1.73 (1.67, 1.80) <0.001	1.74 (1.67, 1.80) <0.001	1.30 (1.25, 1.35) <0.001
Age (years)			
<40	2.42 (2.27, 2.57) < 0.001	2.22 (2.07, 2.37) < 0.001	1.44 (1.34, 1.55) < 0.001
≥ 40	1.92 (1.85, 2.00) <0.001	1.92 (1.85, 2.00) <0.001	1.32 (1.26, 1.37) <0.001
BMI (kg/m2)			
<25	1.84 (1.76, 1.91) < 0.001	1.63 (1.56, 1.70) <0.001	1.34 (1.29, 1.40) <0.001
≥ 25	1.54 (1.45, 1.64) <0.001	1.47 (1.38, 1.57) <0.001	1.30 (1.22, 1.38) <0.001
TG (mmol/L)			
<1.7	3.53 (3.33, 3.75) < 0.001	3.30 (3.10, 3.52) <0.001	1.84 (1.70, 1.99) <0.001
≥ 1.7	1.17 (1.11, 1.23) <0.001	1.15 (1.09, 1.21) <0.001	1.08 (1.02, 1.14) <0.001
Hypertension			
No	2.05 (1.98, 2.12) <0.001	1.89 (1.82, 1.96) <0.001	1.35 (1.30, 1.41) <0.001
Yes	1.84 (1.65, 2.05) <0.001	1.71 (1.53, 1.90) <0.001	1.71 (1.53, 1.90) <0.001
WC (cm)			
Normal	1.96 (1.89, 2.03) <0.001	1.69 (1.63, 1.76) <0.001	1.51 (1.45, 1.57) <0.001
Elevated	1.89 (1.75, 2.03) <0.001	1.58 (1.46, 1.71) <0.001	1.58 (1.46, 1.71) <0.001

Model 1: No covariates were adjusted. Model 2: age and sex were adjusted. Model 3: age, sex, hypertension, smoking status, drinking status, exercise status, WC, HbA1c, ALT, AST, GGT, and LDL-C were adjusted. In the subgroup analysis for sex, the model was not adjusted for sex. In the subgroup analysis for age, the model was not adjusted for age. In the subgroup analysis for hypertension, the model was not adjusted for hypertension. In the subgroup analysis for WC, the model was not adjusted for WC. Elevated WC was defined as ≥ 90 cm in men and ≥ 80 cm in women; normal WC was defined as < 90 cm in men and < 80 cm in women. The first TyHGB quartile contained only four MASLD cases, leading to quasi-complete separation in standard logistic regression. The large quartile odds ratios are susceptible to sparse-data bias and should be interpreted with caution. MASLD, Metabolic dysfunction associated steatotic liver disease; TyHGB, Triglyceride high-density cholesterol-glucose body index; BMI, Body mass index; WC, Waist circumference; HbA1c, Glycosylated hemoglobin; ALT, Alanine aminotransferase; AST, Aspartate aminotransferase; GGT, Gamma-glutamyl transpeptidase; TG, Triglycerides; LDL-C, Low-density lipoprotein cholesterol; OR, Odds ratios; CI, Confidence interval.

When TyHGB was stratified into quartiles, participants in the highest quartile (Q4) exhibited a markedly higher prevalence of MASLD compared to those in the lowest quartile (Q1). However, given the extremely low event count in the reference group (Q1, *n* = 4, as shown in Table 1), the point estimates for the odds ratios (e.g., OR = 65.79 for Q4 in Model 3) are susceptible to sparse data bias and should be interpreted with caution. We therefore primarily emphasize the robust dose-response trend (*P* for trend < 0.001) and the significant association observed in the continuous variable analysis (OR = 1.35 per 1-unit increase in Model 3), which provides a more stable and reliable estimate of the association magnitude.

Stratified subgroup analyses were further conducted by sex, age, BMI, TG, hypertension status, and WC. In men, TyHGB was strongly associated with MASLD risk across all models (Model 1: OR = 2.86, 95% CI: 2.63–3.11; Model 2: OR = 2.79, 95% CI: 2.56–3.04; Model 3: OR = 1.73, 95% CI: 1.57–1.90). A comparable significant association was found in women (Model 1: OR = 1.73, 95% CI: 1.67–1.80; Model 2: OR = 1.74, 95% CI: 1.67–1.80; Model 3: OR = 1.30, 95% CI: 1.25–1.35).

By age grouping, the positive association remained significant in participants aged < 40 years (Model 1: OR = 2.42, 95% CI: 2.27–2.57; Model 2: OR = 2.22, 95% CI: 2.07–2.37; Model 3: OR = 1.44, 95% CI: 1.34–1.55) and those aged ≥ 40 years (Model 1: OR = 1.92, 95% CI: 1.85–2.00; Model 2: OR = 1.92, 95% CI: 1.85–2.00; Model 3: OR = 1.32, 95% CI: 1.26–1.37).

For BMI subgroups, significant positive associations were detected in both BMI < 25 kg/m^2^ (Model 3: OR = 1.34, 95% CI: 1.29–1.40) and BMI ≥ 25 kg/m^2^ (Model 3: OR = 1.30, 95% CI: 1.22–1.38). For TG levels, consistent positive correlations were observed in TG < 1.7 mmol/L (Model 3: OR = 1.84, 95% CI: 1.70–1.99) and TG ≥ 1.7 mmol/L (Model 3: OR = 1.08, 95% CI: 1.02–1.14).

Irrespective of hypertension status, TyHGB was positively linked to MASLD risk in normotensive (Model 3: OR = 1.35, 95% CI: 1.30–1.41) and hypertensive participants (Model 3: OR = 1.71, 95% CI: 1.53–1.90). WC stratification demonstrated similar findings, with significant positive associations in both normal WC (Model 3: OR = 1.51, 95% CI: 1.45–1.57) and elevated WC groups (Model 3: OR = 1.58, 95% CI: 1.46–1.71).

Collectively, these subgroup analyses demonstrated that the positive association between TyHGB and MASLD risk was consistent and robust across major demographic and clinical subgroups.

### Association between TyHGB and WC

Linear regression was used to examine the association between TyHGB and WC, with results shown in Table 3. TyHGB was positively and independently associated with WC in all three models (all *P* < 0.001). In unadjusted Model 1, each 1-unit increase in TyHGB was related to a β coefficient of 2.70 (95% CI: 2.63–2.76). After adjustment for age and sex in Model 2, the association remained significant (β = 2.08, 95% CI: 2.01–2.15). In Model 3 with full adjustment for confounders, including age, sex, hypertension, lifestyle factors, HbA1c, liver enzymes, and lipid parameters, the β value was 1.64 (95% CI: 1.57–1.71).

**TABLE 3:** Association between TyHGB and WC.

Variable	Model 1 β (95% CI), *P*-value	Model 2 β (95% CI), *P*-value	Model 3 β (95% CI), *P*-value
TyHGB	2.70 (2.63, 2.76) < 0.001	2.08 (2.01, 2.15) < 0.001	1.64 (1.57, 1.71) < 0.001
TyHGB quartile			
Q1(3.90-6.22)	Reference	Reference	Reference
Q2(6.22-6.93)	5.01 (4.68, 5.33) < 0.001	4.13 (3.81, 4.46) < 0.001	3.83 (3.51, 4.14) < 0.001
Q3(6.93-8.05)	10.17 (9.85, 10.49) < 0.001	8.40 (8.05, 8.75) < 0.001	7.56 (7.21, 7.91) < 0.001
Q4(8.05-44.03)	16.44 (16.12, 16.76) < 0.001	14.06 (13.69, 14.43) < 0.001	12.06 (11.66, 12.46) < 0.001
*P* for trend	<0.001	< 0.001	<0.001

Model 1: No covariates were adjusted. Model 2: age and sex were adjusted. Model 3: age, sex, hypertension, smoking status, drinking status, exercise status, HbA1c, ALT, AST, GGT, and LDL-C were adjusted. TyHGB, Triglyceride high-density cholesterol-glucose body index; WC, Waist circumference; HbA1c, Glycosylated hemoglobin; ALT, Alanine aminotransferase; AST, Aspartate aminotransferase; GGT, Gamma-glutamyl transpeptidase; LDL-C, Low-density lipoprotein cholesterol; β, Standardized regression coefficient; CI, Confidence interval.

When stratified by TyHGB quartiles with Q1 as the reference group, a significant linear trend was observed between TyHGB and WC (all *P* for trend < 0.001). In the fully adjusted model, the β estimates were 3.83 (95% CI: 3.51–4.14) for Q2, 7.56 (95% CI: 7.21–7.91) for Q3, and 12.06 (95% CI: 11.66–12.46) for Q4.

### Relationship between WC and MASLD

Multivariate logistic regression analyses were conducted to evaluate the independent association between WC and MASLD risk, with detailed results presented in Table 4. WC was significantly and positively associated with the risk of MASLD in all three regression models (all *P* < 0.001). In the unadjusted Model 1, each 1-unit increment in WC was associated with an OR of 1.24 (95% CI: 1.23–1.25). Following adjustment for age and sex in Model 2, the positive association remained robust, with an OR of 1.23 (95% CI: 1.22–1.24). In the fully adjusted Model 3, the OR was 1.19 (95% CI: 1.18–1.20), confirming that WC was independently linked to elevated MASLD risk.

**TABLE 4:** Association between WC and the MASLD risk.

Variable	Model 1 OR (95% CI), *P-*value	Model 2 OR (95% CI), *P-*value	Model 3 OR (95% CI), *P-*value
WC (cm)	1.24 (1.23, 1.25) < 0.001	1.23 (1.22, 1.24) < 0.001	1.19 (1.18, 1.20) < 0.001
WC quartile (cm)			
Q1 (49.00–69.00)	Reference	Reference	Reference
Q2 (69.00–75.50)	21.96 (6.92, 69.74) < 0.001	18.11 (5.70, 57.57) < 0.001	15.41 (4.84, 49.13) < 0.001
Q3 (75.50–82.00)	145.18 (46.58, 452.43) < 0.001	104.52 (33.46, 326.52) < 0.001	70.85 (22.62, 221.92) < 0.001
Q4 (82.00–130.00)	916.16 (294.78, 2847.35) < 0.001	617.99 (198.23, 1926.58) < 0.001	302.85 (96.88, 946.76) < 0.001
*P* for trend	<0.001	< 0.001	<0.001

Model 1: No covariates were adjusted. Model 2: age and sex were adjusted. Model 3: age, sex, hypertension, smoking status, drinking status, exercise status, HbA1c, ALT, AST, GGT, and LDL-C were adjusted. The lowest WC quartile had extremely sparse MASLD events, resulting in unstable point estimates and inflated odds ratios due to quasi-complete separation. Caution is needed when interpreting discrete quartile effect sizes. MASLD, Metabolic dysfunction associated steatotic liver disease; WC, Waist circumference; HbA1c, Glycosylated hemoglobin; ALT, Alanine aminotransferase; AST, Aspartate aminotransferase; GGT, Gamma-glutamyl transpeptidase; LDL-C, Low-density lipoprotein cholesterol; OR, Odds ratios; CI, Confidence interval.

### Non-linear relationship analysis

**FIGURE 2:**
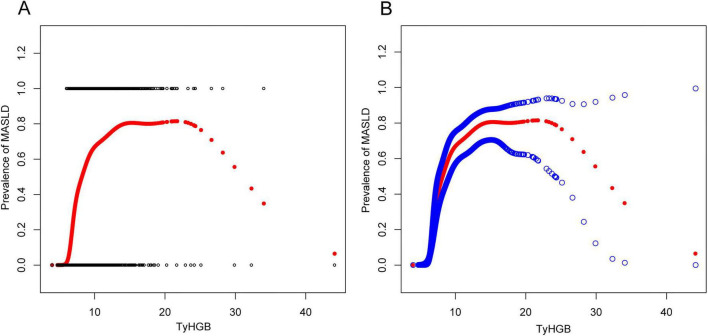
Associations between TyHGB and prevalence of MASLD. **(A,B)** Associations between TYHGB and prevalence of MASLD. The solid red line represents the smooth curve fit between variables. Blue bands represent the 95% confidence interval from the fit. They were adjusted for age, sex, hypertension, smoking status, drinking status, exercise status, HbA1c, WC, ALT, AST, GGT, and LDL-C. MASLD, Metabolic dysfunction-associated steatotic liver disease; TyHGB, Triglyceride high-density cholesterol-glucose body index; WC, Waist circumference; HbA1c, Glycosylated hemoglobin; ALT, Alanine aminotransferase; AST, Aspartate aminotransferase; GGT, Gamma-glutamyl transpeptidase; LDL-C, Low-density lipoprotein cholesterol.

**FIGURE 3:**
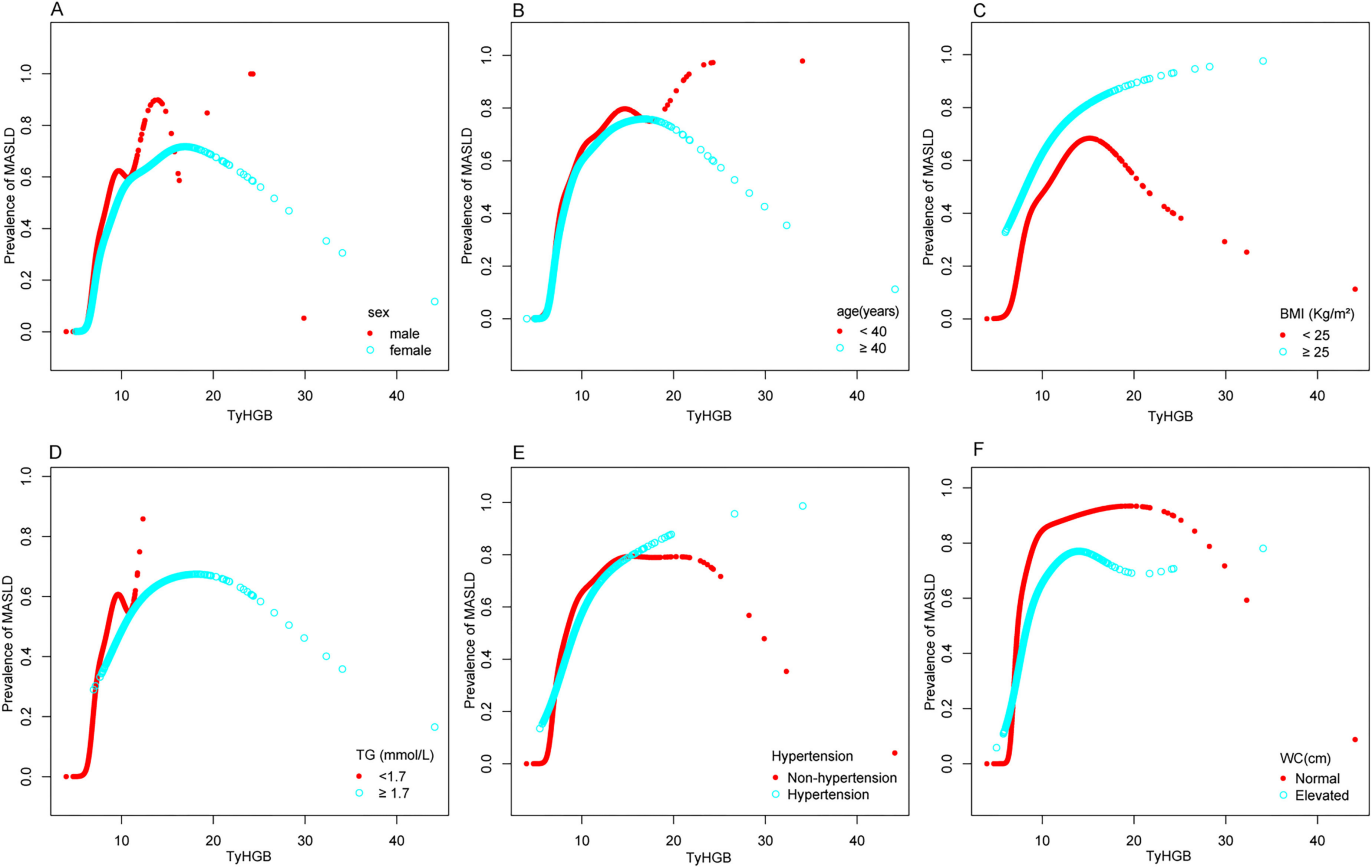
Associations between TyHGB and the prevalence of MASLD by sex **(A)**, age **(B)**, BMI **(C)**, TG **(D)**, hypertension **(E)**, and WC **(F)**. They were adjusted for age, sex, hypertension, smoking status, drinking status, exercise status, HbA1c, WC, ALT, AST, GGT, and LDL-C. In subgroup analyses, the model was not adjusted for the classified variables. MASLD, Metabolic dysfunction-associated steatotic liver disease; TyHGB, Triglyceride high-density cholesterol-glucose body index; BMI, Body mass index; WC, Waist circumference; HbA1c, Glycosylated hemoglobin; ALT, Alanine aminotransferase; AST, Aspartate aminotransferase; GGT, Gamma-glutamyl transpeptidase; TG, Triglycerides; LDL-C, Low-density lipoprotein cholesterol.

We performed smooth curve fitting to evaluate the non-linear association between TyHGB and MASLD. A positive, non-linear correlation was observed between TyHGB and MASLD risk in the overall study population (Figure 2). Subgroup analyses stratified by sex, age, BMI, TG levels, hypertension status, and WC also revealed consistent non-linear trends across all subgroups (Figure 3).

Two-piecewise linear regressions were performed to identify the inflection point of TyHGB linked to MASLD prevalence (Table 5). The overall inflection point for the whole cohort was 9.70: TyHGB below 9.70 yielded a strongly significant positive association with MASLD (OR = 2.02, *P* < 0.001), while the correlation turned non-significant above this cutoff (OR = 1.04, *P* = 0.090). However, the sample distribution was heavily weighted below the inflection point (*n* = 12,397) compared to above it (*n* = 1,285), as detailed in Supplementary Table 4. This data sparsity in the upper tail may contribute to visual fluctuations in the smoothed curves, despite the regression analysis indicating a lack of significant continued risk elevation.

**TABLE 5:** Threshold effect analysis of TyHGB on the prevalence of MASLD across sex, age, BMI, TG, hypertension, and WC using a two-piecewise linear regression model.

Prevalence of MASLD	Adjusted OR (95% CI), *P*-value
All participants	
Fitting by the standard linear model	1.35 (1.31, 1.40), < 0.001
Fitting by the two-piecewise linear model	
Inflection point	9.70
TyHGB < 9.70	2.02 (1.88, 2.17), < 0.001
TyHGB > 9.70	1.04 (0.99, 1.09), 0.090
Log likelihood ratio	<0.001
Men	
Fitting by the standard linear model	1.73 (1.57, 1.90), < 0.001
Fitting by the two-piecewise linear model	
Inflection point	7.43
TyHGB < 7.43	8.56 (5.37, 13.65), < 0.001
TyHGB > 7.43	1.31 (1.17, 1.46), < 0.001
Log likelihood ratio	<0.001
Women	
Fitting by the standard linear model	1.11 (1.06, 1.16), < 0.001
Fitting by the two-piecewise linear model	
Inflection point	14.51
TyHGB < 14.51	1.23 (1.15, 1.32), < 0.001
TyHGB > 14.51	0.93 (0.84, 1.04), 0.191
Log likelihood ratio	<0.001
Age < 40 (years)	
Fitting by the standard linear model	1.44 (1.34, 1.55), < 0.001
Fitting by the two-piecewise linear model	
Inflection point	7.22
TyHGB < 7.22	15.40 (6.75, 35.13), < 0.001
TyHGB > 7.22	1.30 (1.21, 1.40), < 0.001
Log likelihood ratio	<0.001
Age ≥ 40 (years)	
Fitting by the standard linear model	1.07 (1.02, 1.13), 0.006
Fitting by the two-piecewise linear model	
Inflection point	14.75
TyHGB < 14.75	1.20 (1.11, 1.29), < 0.001
TyHGB > 14.75	0.90 (0.80, 1.01), 0.082
Log likelihood ratio	<0.001
BMI < 25 (kg/m2)	
Fitting by the standard linear model	1.34 (1.29, 1.40), < 0.001
Fitting by the two-piecewise linear model	
Inflection point	8.53
TyHGB < 8.53	2.99 (2.59, 3.46), < 0.001
TyHGB > 8.53	1.09 (1.04, 1.14), < 0.001
Log likelihood ratio	< 0.001
BMI ≥ 25 (kg/m2)	
Fitting by the standard linear model	1.30 (1.22, 1.38), < 0.001
Fitting by the two-piecewise linear model	
Inflection point	10.24
TyHGB < 10.24	1.50 (1.34, 1.67), < 0.001
TyHGB > 10.24	1.12 (1.01, 1.24), 0.030
Log likelihood ratio	0.002
TG < 1.7 (mmol/L)	
Fitting by the standard linear model	1.85 (1.71, 2.00), < 0.001
Fitting by the two-piecewise linear model	
Inflection point	7.20
TyHGB < 7.20	13.01 (8.05, 21.02), < 0.001
TyHGB > 7.20	1.47 (1.35, 1.61), < 0.001
Log likelihood ratio	< 0.001
TG ≥ 1.7 (mmol/L)	
Fitting by the standard linear model	1.07 (1.01, 1.13),0.022
Fitting by the two-piecewise linear model	
Inflection point	14.51
TyHGB < 14.51	1.21 (1.10, 1.34), < 0.001
TyHGB > 14.51	0.94 (0.85, 1.05), 0.273
Log likelihood ratio	0.002
Non-Hypertension	
Fitting by the standard linear model	1.11 (1.06, 1.16), < 0.001
Fitting linear model by the two-piecewise	
Inflection point	14.35
TyHGB < 14.35	1.24 (1.15, 1.33), < 0.001
TyHGB > 14.35	0.95 (0.85, 1.05), 0.273
Log likelihood ratio	<0.001
Hypertension	
Fitting by the standard linear model	1.36 (1.21, 1.53), < 0.001
Fitting by the two-piecewise linear model	
Inflection point	7.54
TyHGB < 7.54	5.52 (1.82, 16.78), 0.026
TyHGB > 7.54	1.26 (1.12, 1.43), < 0.001
Log likelihood ratio	<0.001
Normal WC	
Fitting by the standard linear model	1.13 (1.08, 1.18), < 0.001
Fitting by the two-piecewise linear model	
Inflection point	14.62
TyHGB < 14.62	1.25 (1.16, 1.34), < 0.001
TyHGB > 14.62	0.96 (0.86, 1.07), 0.4410
Log likelihood ratio	<0.001
Elevated WC	
Fitting by the standard linear model	1.42 (1.30, 1.54), < 0.001
Fitting by the two-piecewise linear model	
Inflection point	8.97
TyHGB < 8.97	2.36 (1.98, 2.82), < 0.001
TyHGB > 8.97	1.08 (0.98, 1.19), 0.101
Log likelihood ratio	<0.001

Age, sex, hypertension status, smoking status, drinking status, exercise status, WC, HbA1c, ALT, AST, GGT, and LDL-C were adjusted for. In the subgroup analysis for sex, the model was not adjusted for sex. In the subgroup analysis for age, the model was not adjusted for age. In the subgroup analysis for hypertension, the model was not adjusted for hypertension. In the subgroup analysis for WC, the model was not adjusted for WC. Elevated WC was defined as ≥ 90 cm in men and ≥ 80 cm in women; normal WC was defined as < 90 cm in men and < 80 cm in women. MASLD, Metabolic dysfunction associated steatotic liver disease; TyHGB, Triglyceride high-density cholesterol-glucose body index; BMI, Body mass index; WC, Waist circumference; HbA1c, Glycosylated hemoglobin; ALT, Alanine aminotransferase; AST, Aspartate aminotransferase; GGT, Gamma-glutamyl transpeptidase; TG, Triglycerides; LDL-C, Low-density lipoprotein cholesterol; OR, Odds ratios; CI, Confidence interval.

Subgroup-stratified models revealed distinct inflection thresholds separated by metabolic susceptibility. Subgroups with inherently higher metabolic vulnerability exhibited lower inflection points: 7.43 for men, 7.22 for participants aged < 40 years, 7.20 for those with TG < 1.7 mmol/L, 7.54 for hypertensive patients, and 8.97 for individuals with elevated WC. By contrast, metabolically resilient subgroups displayed markedly higher inflection cutoffs: 14.51 for women, 14.75 for adults aged ≥ 40 years, 10.24 for participants with BMI ≥ 25 kg/m^2^, 14.51 for subjects with TG ≥ 1.7 mmol/L, 14.35 for normotensive individuals, and 14.62 for those with normal WC. Log-likelihood ratio tests all returned *P* < 0.001 across all strata, verifying that two-piecewise regression models fitted the data significantly better than standard single linear regression.

### A ROC analysis of TyHGB as a MASLD predictor

**FIGURE 4:**
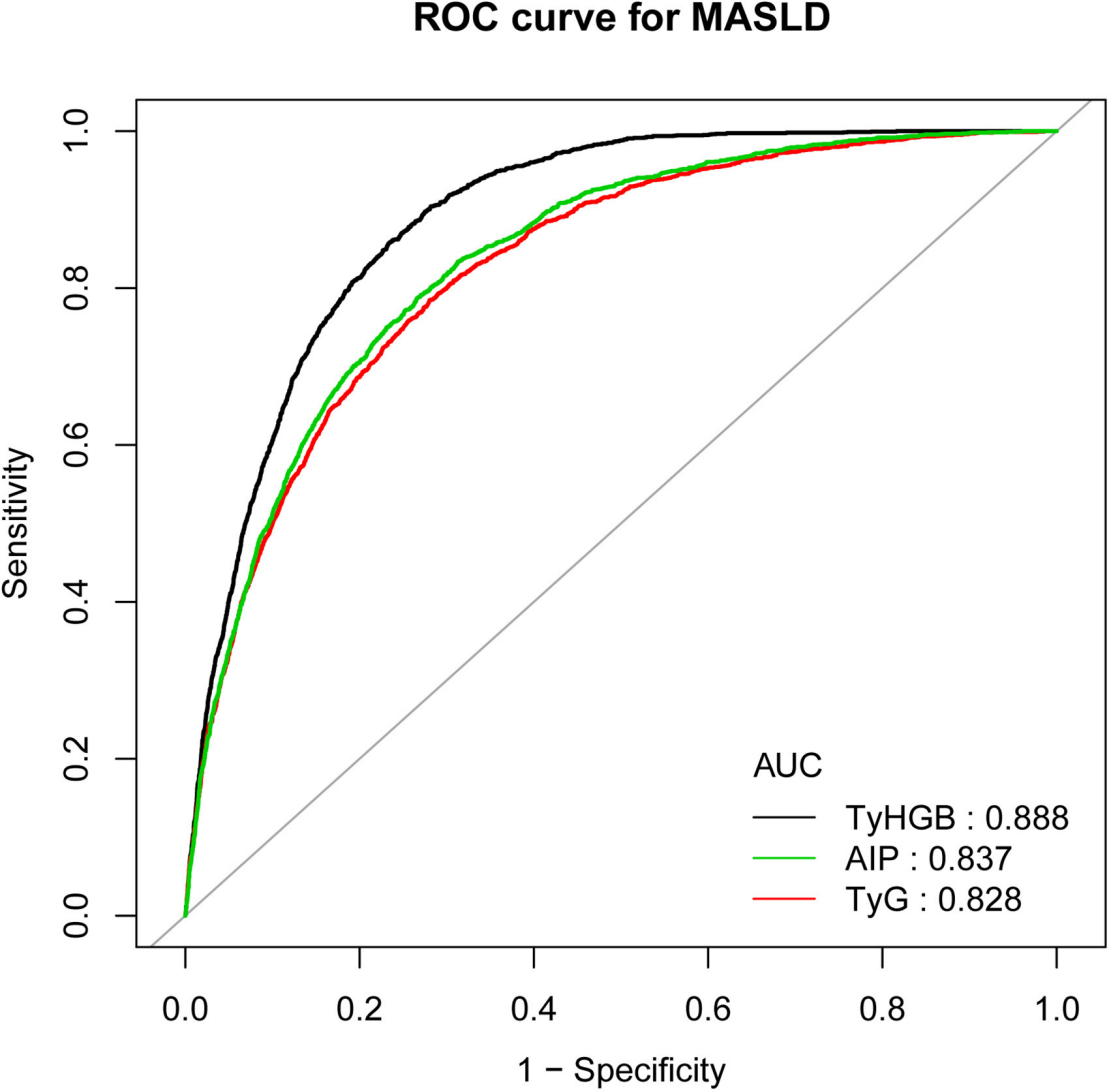
ROC curves comparing the predictive value of TyHGB with TyG, and AIP for MASLD onset, with predictive efficacy assessed by AUC in the general population. MASLD, Metabolic dysfunction-associated steatotic liver disease; TyHGB, Triglyceride High-Density Cholesterol-Glucose Body Index; AIP, Atherogenic index of plasma; TyG, Triglyceride-glucose; ROC, Receiver operating characteristic; AUC, Area under the curve.

**FIGURE 5:**
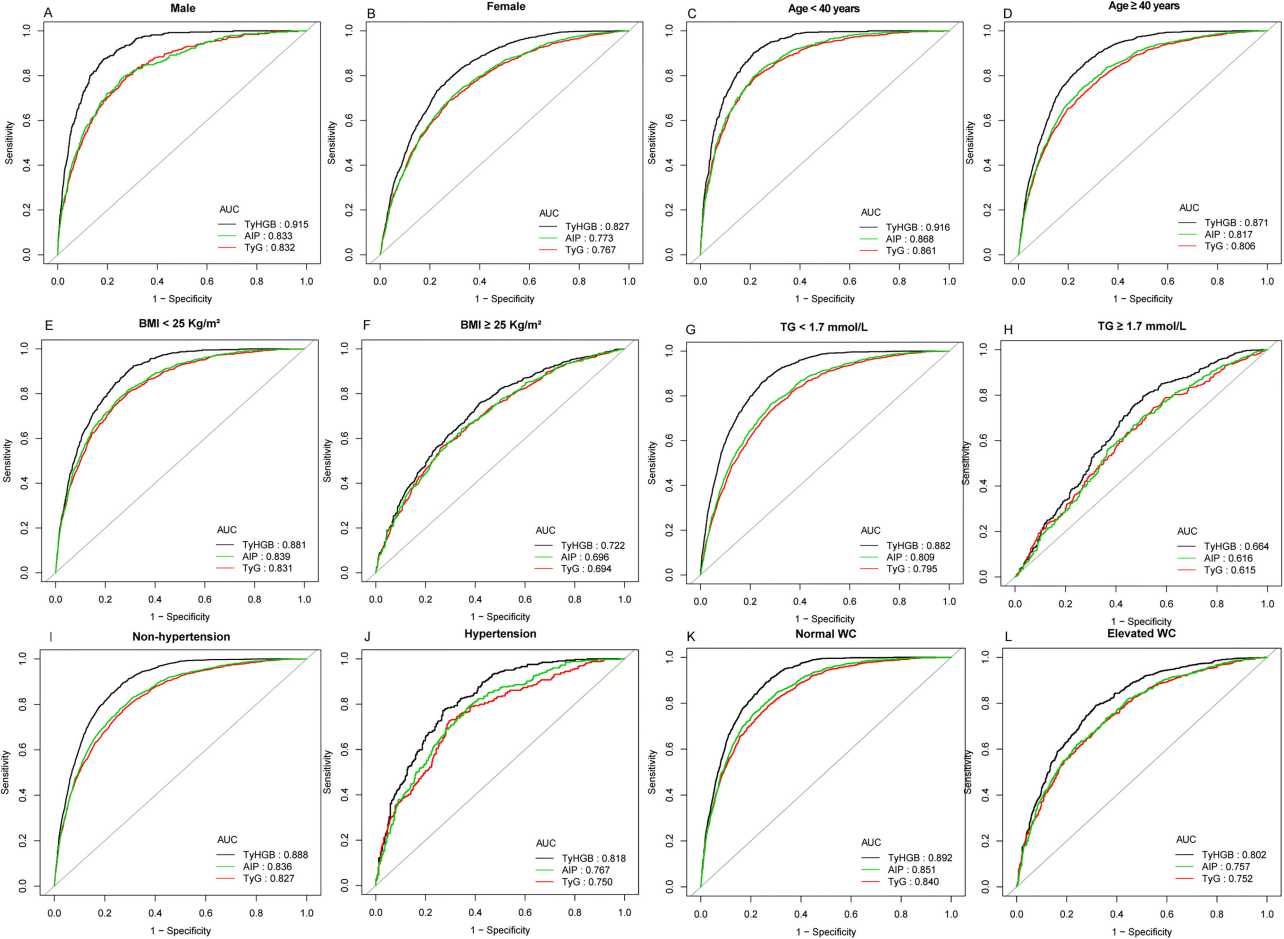
ROC curves comparing the predictive value of TyHGB with TyG, and AIP for MASLD onset, with predictive efficacy assessed by AUC. **(A,B)** Sex stratification: TyHGB outperformed TyG and AIP in males **(A)** and females **(B)**. **(C,D)** Age stratification: TyHGB showed better predictive performance in subjects < 40 years **(C)** and ≥ 40 years **(D)**. **(E,F)** BMI stratification: TyHGB showed higher predictive efficacy in BMI < 25 kg/m^2^ **(E)** and ≥ 25 kg/m^2^ **(F)**. **(G,H)** TG stratification: TyHGB outperformed TyG, and AIP in TG < 1.7 mmol/L **(G)** and ≥ 1.7 mmol/L **(H)**. **(I,J)** Hypertension stratification: TyHGB outperformed TyG and AIP in non-hypertension **(I)** and hypertension **(J)**. **(K,L)** WC stratification: TyHGB outperformed TyG and AIP in Normal WC **(K)** and Elevated WC **(L)**. MASLD, Metabolic dysfunction-associated steatotic liver disease; Triglyceride high-density cholesterol-glucose body index; BMI, Body mass index; WC, Waist circumference; TG, Triglycerides; AIP, Atherogenic index of plasma; TyG, Triglyceride-glucose; ROC, Receiver operating characteristic; AUC, Area under the curve. Elevated WC was defined as ≥ 90 cm in men and ≥ 80 cm in women; normal WC was defined as < 90 cm in men and < 80 cm in women.

Receiver operating characteristic (ROC) analysis was performed to assess the predictive performance of TyHGB for MASLD, with the triglyceride-glucose index (TyG) and the atherogenic index of plasma (AIP) as comparative indicators (Figures 4, 5; Supplementary Table 1). In the overall study population, TyHGB showed the highest diagnostic value, with an area under the curve (AUC) of 0.8882 (95% CI: 0.8819–0.8945), which was significantly higher than that of TyG (0.8279) and AIP (0.8369). The optimal cutoff value of TyHGB was 7.5974, with a sensitivity of 0.8577 and a specificity of 0.7661.

We further conducted stratified ROC analyses across key subgroups (Figure 5). In sex-stratified analysis, TyHGB remained superior to TyG and AIP in both men and women. In men, the AUC of TyHGB was 0.9155 (95% CI: 0.9046–0.9264), with a cutoff of 7.1131, sensitivity of 0.8740, and specificity of 0.8160. In women, the AUC was 0.8272 (95% CI: 0.8168–0.8375), with a cutoff of 8.0235, sensitivity of 0.8027, and specificity of 0.7071.

For age subgroups, TyHGB demonstrated an AUC of 0.9157 (95% CI: 0.9067–0.9247) in participants aged < 40 years and 0.8707 (95% CI: 0.8622–0.8792) in those aged ≥ 40 years. Regarding BMI categories, the AUC values were 0.8809 (95% CI: 0.8717–0.8901) in the BMI < 25 kg/m^2^ group and 0.7222 (95% CI: 0.7006–0.7438) in the BMI ≥ 25 kg/m^2^ group.

In TG-stratified analysis, the AUC of TyHGB was 0.8817 (95% CI: 0.8743–0.8891) in participants with TG < 1.7 mmol/L and 0.6642 (95% CI: 0.6324–0.6959) in those with TG ≥ 1.7 mmol/L. Similarly, TyHGB maintained robust predictive performance for MASLD across subgroups stratified by hypertension status and WC. In all subgroups, the AUC of TyHGB was consistently higher than that of TyG and AIP (all *P* < 0.01).

### The potential mediating role of WC in the relationship between TyHGB and MASLD

**FIGURE 6:**
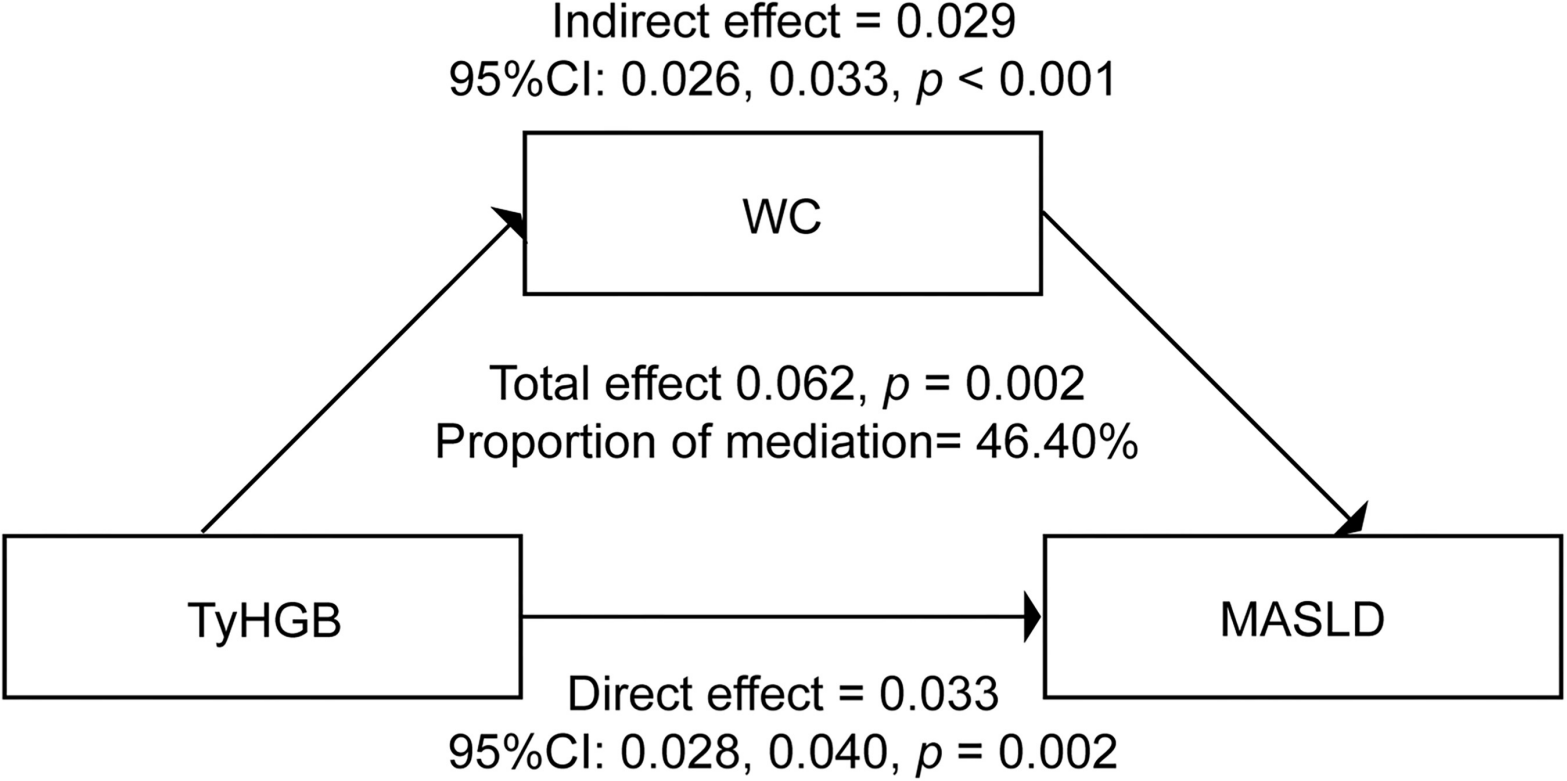
The mediation effect of WC between TyHGB and MASLD in the general population. Adjusted for age, sex, hypertension, smoking status, drinking status, exercise status, HbA1c, ALT, AST, GGT, and LDL-C. MASLD, Metabolic dysfunction-associated steatotic liver disease; TyHGB, Triglyceride high-density cholesterol-glucose body index; WC, Waist circumference; HbA1c, Glycosylated hemoglobin; ALT, Alanine aminotransferase; AST, Aspartate aminotransferase; GGT, Gamma-glutamyl transpeptidase; TG, Triglycerides; LDL-C, Low-density lipoprotein cholesterol.

Mediation models were constructed to examine whether WC may serve as a potential mediator of the association between the TyHGB index and MASLD (Figure 6; Supplementary Table 2). All models were adjusted for age, sex, hypertension, smoking, drinking, exercise status, HbA1c, ALT, AST, GGT, TG, and LDL-C.

In the overall study subjects, TyHGB showed a total effect on MASLD risk of 0.062 (95% CI 0.056–0.071, *P* = 0.002). A significant indirect effect through WC was estimated at 0.029 (95% CI 0.026–0.033, *P* < 0.001), accompanied by a direct effect of 0.033 (95% CI 0.028–0.040, *P* = 0.002). These results suggested that approximately 46.40% of the association between TyHGB and MASLD could be potentially explained by WC (*P* = 0.002).

**FIGURE 7:**
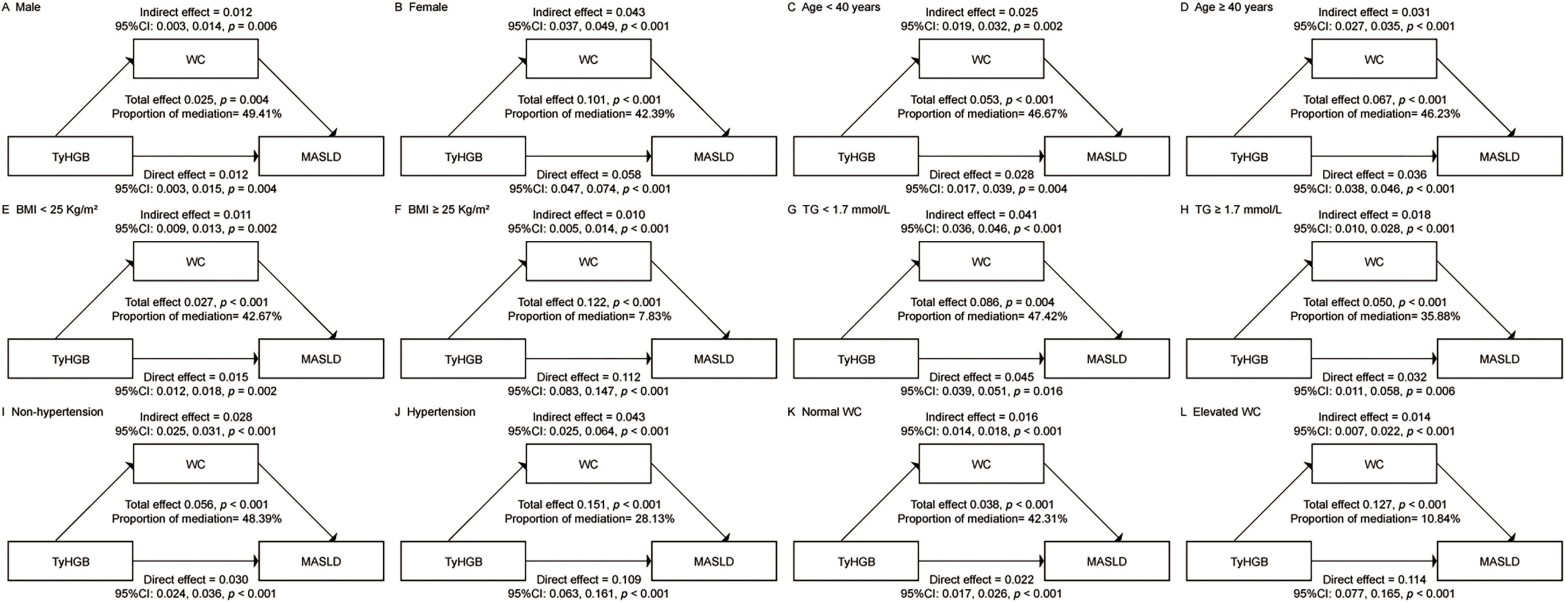
The mediation effect of WC between TyHGB and MASLD by sex **(A,B)**, age **(C,D)**, BMI **(E,F)**, TG **(G,H)**, hypertension **(I,J)**, and WC **(K,L)**. Adjusted for age, sex, hypertension, smoking status, drinking status, exercise status, HbA1c, ALT, AST, GGT, and LDL-C. In subgroup analyses, the model was not adjusted for the classified variables. MASLD, Metabolic dysfunction-associated steatotic liver disease; TyHGB, Triglyceride high-density cholesterol-glucose body index; BMI, Body mass index; WC, Waist circumference; HbA1c, Glycosylated hemoglobin; ALT, Alanine aminotransferase; AST, Aspartate aminotransferase; GGT, Gamma-glutamyl transpeptidase; TG, Triglycerides; LDL-C, Low-density lipoprotein cholesterol. Elevated WC was defined as ≥ 90 cm in men and ≥ 80 cm in women; normal WC was defined as < 90 cm in men and < 80 cm in women.

Stratified analyses were further conducted to assess the consistency of this potential mediating pathway across subgroups (Figure 7; Supplementary Table 2). WC appeared to mediate the link between TyHGB and MASLD in both men and women, with corresponding mediated proportions of 49.41 and 42.39%, respectively. Similar patterns of potential mediation were observed across age strata, with mediated proportions of 46.67% in participants younger than 40 years and 46.23% in those aged 40 years or older.

Notably, the strength of this potential mediation appeared to vary by metabolic status. The mediated proportion was 42.67% in participants with BMI < 25 kg/m^2^, but declined to only 7.83% in those with BMI ≥ 25 kg/m^2^. Likewise, the proportion mediated by WC was 47.42% in participants with TG < 1.7 mmol/L and 35.88% in those with TG ≥ 1.7 mmol/L. With respect to hypertension, WC potentially accounted for 48.39% of the association in normotensive individuals but only 28.13% in hypertensive participants. For baseline WC categories, the mediated proportion was 42.31% in participants with normal WC and merely 10.84% in those with elevated WC.

Collectively, these findings support that WC may play a partial and potential mediating role in the relationship between TyHGB and MASLD. While this potential mediation was generally consistent across subgroups, its magnitude was notably attenuated among individuals with adverse metabolic profiles.

### Sensitivity analysis addressing potential mathematical coupling

When using the modified index TyHGB_noBMI (excluding the BMI term) as the exposure, the total effect on MASLD remained significant (total effect = 0.055, 95% CI 0.049–0.063). WC mediated approximately 40.44% of this association (average mediation effect = 0.022, 95% CI 0.020–0.025; proportion mediated = 0.404, 95% CI 0.360–0.448). The direct effect also remained significant (average direct effect = 0.033, 95% CI 0.028–0.039). Detailed results are presented in Supplementary Table 3.

## Discussion

In this cross-sectional analysis of 13,682 non-diabetic Japanese adults, we characterized the association between TyHGB and MASLD. TyHGB was independently associated with higher MASLD risk, with a non-linear pattern and a clear inflection point at 9.70. Below this threshold, the risk gradient was steep (OR = 2.02); above it, the incremental association plateaued. Waist circumference partially mediated 46.4% of the overall TyHGB–MASLD association, but this mediating effect differed markedly by metabolic status, reaching 42.67% in normal-weight individuals. Still, it dropped to 7.83% in obese participants. TyHGB also showed better discriminative performance (AUC = 0.888) than TyG (0.828) and AIP (0.837). These findings suggest that TyHGB may be particularly valuable for identifying occult MASLD in non-obese populations, where WC captures a larger share of the risk pathway.

To date, only one existing study has explored the association of TyHGB with NAFLD, with relevant evidence largely sourced from Chinese populations and the US NHANES cohort (34). Unlike earlier work, our analysis adopted the updated global MASLD criteria, which better capture the metabolism-driven pathological features of hepatic steatosis. A major design advantage of our study is the strict exclusion of individuals with baseline diabetes, an approach that lessens confounding from diabetic status and hypoglycemic therapy on glucose–lipid profiles, body composition, and hepatic fat deposition. This enables a more reliable evaluation of the intrinsic link between TyHGB-related metabolic dysregulation and early-stage hepatic steatosis. Beyond simple association assessment, we further quantified the mediating role of abdominal obesity and its subgroup disparities, which may partially interpret the robust association observed in fully adjusted models (OR = 1.35). Accumulating evidence also links TyHGB to multiple metabolic conditions, including gestational diabetes mellitus, diabetic nephropathy, ischemic stroke, gout, hyperuricemia, and coronary artery disease (29, 30, 33, 36, 37). Collectively, TyHGB appears to reflect underlying atherogenic metabolic dysfunction closely tied to IR, and may serve as a shared metabolic correlate across chronic metabolic disorders, including MASLD.

It is important to note that the observed “mediation” reflects a statistical decomposition of the association rather than a definitive biological cascade, given the limitations of cross-sectional mediation analysis. However, these findings align with established pathophysiological concepts wherein visceral adiposity triggers lipolysis and releases free fatty acids (FFAs) into the portal circulation, providing abundant substrates for intrahepatic triglyceride synthesis (38, 39). Concurrent adipokine dysregulation (e.g., reduced adiponectin and elevated leptin) may potentiate inflammatory signaling (e.g., NF-κB/TLR4 pathways) and oxidative stress, thereby exacerbating hepatic insulin resistance (21, 40–44). While our cross-sectional design cannot dissect these cellular mechanisms, the observed statistical mediation by WC suggests that such metabolic and inflammatory pathways likely contribute to the TyHGB–MASLD link.

A potential limitation in interpreting this mediation effect is the high collinearity between BMI (a component of TyHGB) and WC. To mitigate concerns regarding mathematical coupling, we noted that the direct effect of TyHGB remained significant despite adjusting for WC in regression models, suggesting that TyHGB captures metabolic signals beyond simple anthropometric measures. Nevertheless, the quantified mediation proportion should be interpreted as an approximation of the relative contribution of central adiposity within the statistical model. To further address concerns regarding mathematical coupling, we performed a sensitivity analysis using TyHGB_noBMI, which excludes the BMI component. The estimated mediated proportion decreased from 46.4% in the primary model to approximately 40% when the BMI term was removed, confirming that a portion of the original mediation estimate was attributable to the shared BMI component and the high correlation between BMI and WC. However, the persistence of a substantial and statistically significant indirect effect (mediated proportion ∼40%) indicates that WC-mediated pathways represent a genuine biological mechanism rather than an algebraic artifact. This result supports the interpretation that TyHGB-associated metabolic dysregulation acts partly through visceral adiposity (WC) and downstream lipotoxicity to promote MASLD, and partly through non-WC pathways (e.g., direct lipotoxicity, systemic inflammation, *de novo* lipogenesis). It also strengthens the conclusion that TyHGB is particularly useful for identifying risk among non-obese individuals, in whom WC captures a larger share of the risk pathway.

Subgroup stratification revealed clear heterogeneity in the mediating contribution of WC across metabolic subgroups. The mediating proportion remained above 40% in non-obese individuals (BMI < 25 kg/m^2^) and normolipidemic participants (TG < 1.7 mmol/L), whereas it fell below 10% among those with obesity or hypertriglyceridemia. In normal-weight and normolipidemic populations, TyHGB-related metabolic dysregulation tends to drive hidden visceral fat gain rather than apparent generalized obesity. This subgroup generally carries lower glycolipid toxicity and milder inflammatory burden, making visceral fat accumulation the dominant pathway linking TyHGB to hepatic steatosis and thereby yielding a higher mediating proportion. By contrast, obese or hypertriglyceridemic individuals harbor livers already burdened by multiple pathological insults, including excess free fatty acid deposition, hyperglycemia-induced *de novo* lipogenesis, oxidative stress, endoplasmic reticulum stress, and gut-derived inflammatory stimuli, collectively contributing to the progression of liver injury (45–50). Under such complex metabolic backgrounds, there appears to be a relative shift toward non-WC-mediated pathways (e.g., direct lipotoxicity, systemic inflammation), thereby reducing the relative mediating effect of visceral fat. Such heterogeneous patterns reflect distinct metabolic mechanisms across different metabolic subgroups, and offer practical clues for clinical risk stratification. In non-obese, normolipidemic groups, TyHGB can help detect hidden visceral adiposity, underscoring the value of targeted abdominal obesity management. For individuals with obesity or dyslipidemia, TyHGB mirrors broader metabolic derangements, requiring combined interventions targeting glycolipid metabolism and chronic inflammation.

Our analysis identified a non-linear association between TyHGB and MASLD risk, with an overall inflection point at 9.70. Below this threshold, MASLD risk increased rapidly. Above this cutoff, the two-piecewise linear regression indicated that the risk increment plateaued, rather than continuing to rise linearly. However, it is important to note that the participant distribution was unbalanced around this threshold, with substantially fewer subjects in the upper stratum. This sparsity in the high TyHGB group may limit the precision of risk estimation at extreme values and could explain the visual fluctuations or downward trend observed in the smoothed curve fitting. Therefore, while the data suggest a plateau in risk elevation, caution is warranted in interpreting the trajectory at extreme TyHGB values due to the limited sample size.

Consistent with this mediation heterogeneity, the threshold effect analysis further revealed divergent risk trajectories across subgroups. The stratified inflection point disparities reflect distinct TyHGB-related hepatic lipotoxic susceptibility among different metabolic phenotypes. Men and young adults exhibited lower inflection values (7.43 and 7.22), largely due to insufficient estrogen-mediated metabolic protection and earlier visceral fat and insulin resistance accumulation. This early risk elevation pattern matches our mediation results, wherein waist circumference accounts for a larger proportion of the disease pathway in these metabolically sensitive populations. In contrast, women and older participants only showed accelerated MAS risk at much higher TyHGB cutoffs (14.51 and 14.75): endogenous estrogen inhibits visceral fat buildup, whereas cumulative lifelong metabolic disorders raise baseline liver disease risk. Similarly, hypertensive patients and those with elevated WC had low inflection points (7.54, 8.97). Pre-existing central obesity and hypertension form a pro-inflammatory, lipotoxic metabolic background, lowering the TyHGB threshold for hepatic steatosis. Normotensive individuals and participants with normal WC possess stronger metabolic buffering capacity and only experience prominent risk growth when TyHGB rises substantially.

The overall inflection point of 9.70 arises from the mixed composition of our cohort, combining early-sensitive low-threshold subgroups and delayed high-threshold phenotypes. Such threshold heterogeneity echoes the subgroup differences observed in WC-mediated effects: visceral adiposity (WC) dominates disease pathways in low-inflection populations, while direct lipotoxicity and other non-WC pathways become predominant under severe metabolic burden, leading to attenuated WC-mediating effects. Clinically, one-size-fits-all TyHGB cutoffs are inappropriate. Lower thresholds (7.20–10.24) are recommended for early screening in lean, young male high-risk populations, whereas higher cutoffs (14.35 + ) should be adopted to identify additional hepatic steatosis risk in older women and metabolically disordered adults.

It is important to acknowledge the inherent structural overlap between TyHGB and the MASLD diagnostic criteria. Since MASLD is defined by the presence of hepatic steatosis accompanied by specific metabolic dysfunctions (e.g., obesity, dyslipidemia, hyperglycemia), and TyHGB is constructed from these exact metabolic components (TG, HDL-C, FBG, BMI), the strong association observed is partially predetermined by the definition itself. This “circularity” explains the high discriminatory power (AUC ∼0.89) but limits the interpretation of TyHGB as an “independent” biological predictor in the strict sense.

However, this structural overlap does not negate TyHGB’s clinical utility. Rather, it validates its design rationale as a “composite surrogate.” Instead of checking multiple disparate metabolic criteria (BMI, WC, TG, glucose) individually, TyHGB consolidates these core risk drivers into a single, continuous index. A high TyHGB value effectively signals that the subject likely meets the metabolic dysfunction thresholds required for MASLD diagnosis. Therefore, TyHGB’s value lies in operational simplification—facilitating rapid, cost-effective risk triage in primary care settings—rather than identifying novel pathophysiological pathways independent of the defining metabolic traits.

This study possesses several methodological strengths in design and statistical strategy. We analyzed data from a large community-based cohort and included only non-diabetic individuals at baseline, effectively minimizing confounding by diabetes status and antihyperglycemic medication use. Such a design enables a more reliable evaluation of MASLD epidemiological features and metabolic correlates, specifically in non-diabetic populations. We also applied the latest internationally recognized MASLD diagnostic criteria, consistent with mainstream academic consensus. Statistically, we integrated non-linear dose–response modeling, threshold effect analysis, mediation estimation, and subgroup comparison, allowing comprehensive quantification of the heterogeneous mediating role of abdominal obesity in the TyHGB–MASLD association. Clinically, TyHGB is derived from routine biochemical and anthropometric measurements, offering the advantages of non-invasiveness, ease of accessibility, and low cost. These features make TyHGB suitable for large-scale community screening and graded MASLD risk assessment.

Our findings must be interpreted in light of several limitations. Foremost, the cross-sectional design precludes causal inference; prospective cohorts are needed to verify the long-term predictive value of TyHGB for incident MASLD. Additionally, hepatic steatosis was assessed by ultrasound, which lacks sensitivity for mild steatosis, potentially leading to an underestimation of the true association strength between TyHGB and early-stage MASLD. Although major confounders were adjusted for, residual bias from unmeasured variables (e.g., dietary habits, physical activity level, genetic background, gut microbiota) cannot be excluded. Meanwhile, the study population was restricted to non-diabetic Japanese adults, so cautious interpretation is required when extrapolating these findings to other ethnic groups and diabetic populations. In addition, serum insulin data were unavailable in the current cohort, preventing a direct head-to-head comparison between TyHGB and gold-standard IR indicators, including the homeostatic model assessment of IR (HOMA-IR). Furthermore, a potential limitation is the sparse data bias in quartile analyses due to the very low prevalence of MASLD in the lowest TyHGB and WC quartiles. This sparse data structure may have led to inflated OR estimates and wide confidence intervals in standard logistic models. Consequently, we focused our interpretation on the significance of the trend test and continuous associations rather than the absolute magnitude of the quartile-specific ORs to ensure the robustness of our conclusions. Crucially, the mediation results reflect statistical rather than definitive biological causality; thus, the mediating effects should be interpreted prudently without overinterpreting causal pathways among TyHGB, abdominal obesity, and MASLD. Finally, due to the shared metabolic components between the TyHGB index and the MASLD definition, the association reflects a structural association rather than a purely independent causal link, which should be considered when interpreting the biological implications of the risk estimates.

## Conclusion

In non-diabetic Japanese adults, TyHGB is independently and non-linearly associated with prevalent MASLD, with WC partially mediating this relationship. The marked attenuation of WC mediation among obese individuals suggests a relative shift towards non-WC-mediated pathways under high metabolic burden. TyHGB showed favorable discriminative performance compared with conventional metabolic markers and may be particularly useful for identifying occult MASLD among non-obese, normolipidemic individuals who are frequently overlooked by BMI-based screening. Given that TyHGB relies solely on routine physical and biochemical examinations, it represents a cost-effective tool for nutritional epidemiology surveillance and opportunistic screening of “lean MASLD” in primary care settings.

## Data Availability

Publicly available datasets were analyzed in this study. This data can be found here: The data supporting the findings of this study are publicly accessible in the Dryad Digital Repository at https://datadryad.org/stash/dataset/doi:10.5061/dryad.8q0p192.

## Footnotes

1
https://doi.org/10.5061/dryad.8q0p192

2
https://www.R-project.org

3
https://www.empowerstats.com

## References

[1] Kalligeros M Vassilopoulos A Vassilopoulos S Victor DW Mylonakis E Noureddin M. Prevalence of steatotic liver disease (MASLD, MetALD, and ALD) in the United States: NHANES 2017-2020. *Clin Gastroenterol Hepatol*. (2024) 22:1330–2.e4. 10.1016/j.cgh.2023.11.003 37949334

[2] Chan KE Koh TJL Tang ASP Quek J Yong JN Tay P et al. Global prevalence and clinical characteristics of metabolic-associated fatty liver disease: a meta-analysis and systematic review of 10 739 607 individuals. *J Clin Endocrinol Metab*. (2022) 107:2691–700. 10.1210/clinem/dgac321 35587339

[3] Gastaldelli A Newsome PN. NAFLD vs MASLD (metabolic dysfunction-associated steatotic liver disease)-why the need for a change of nomenclature? *J Clin Endocrinol Metab*. (2025) 110:e2407–10. 10.1210/clinem/dgaf094 39970128

[4] Quek J Chan KE Wong ZY Tan C Tan B Lim WH et al. Global prevalence of non-alcoholic fatty liver disease and non-alcoholic steatohepatitis in the overweight and obese population: a systematic review and meta-analysis. *Lancet Gastroenterol Hepatol*. (2023) 8:20–30. 10.1016/S2468-1253(22)00317-X 36400097

[5] Tilg H Petta S Stefan N Targher G. Metabolic dysfunction-associated steatotic liver disease in adults: a review. *JAMA*. (2026) 335:163–74. 10.1001/jama.2025.19615 41212550

[6] Younossi ZM Golabi P Paik JM Henry A Van Dongen C Henry L. The global epidemiology of nonalcoholic fatty liver disease (NAFLD) and nonalcoholic steatohepatitis (NASH): a systematic review. *Hepatology*. (2023) 77:1335–47. 10.1097/HEP.0000000000000004 36626630 PMC10026948

[7] Younossi ZM Paik JM Yilmaz Y Kanat M El-Kassas M Al-Busafi SA et al. Two-decade trends in MASLD and its complications in the Middle East and North Africa (2010-2021). *Liver Int*. (2025) 45:e70342. 10.1111/liv.70342 40919841

[8] Eslam M Newsome PN Sarin SK Anstee QM Targher G Romero-Gomez M et al. A new definition for metabolic dysfunction-associated fatty liver disease: an international expert consensus statement. *J Hepatol*. (2020) 73:202–9. 10.1016/j.jhep.2020.03.039 32278004

[9] Chew NWS Mehta A Goh R Koh J Chen Y Chong B et al. The global cardiovascular-liver-metabolic syndemic: epidemiology, trends and challenges. *Nat Rev Cardiol*. (2026) 23:219–38. 10.1038/s41569-025-01220-4 41053364

[10] Zhang GY Brandman DA. Clinical update on MASLD. *JAMA Intern Med*. (2025) 185:105–7. 10.1001/jamainternmed.2024.6431 39585671

[11] Huang YH Chan C Lee HW Huang C Chen YJ Liu PC et al. Influence of nonalcoholic fatty liver disease with increased liver enzyme levels on the risk of cirrhosis and hepatocellular carcinoma. *Clin Gastroenterol Hepatol*. (2023) 21:960–9.e1. 10.1016/j.cgh.2022.01.046 35124270 PMC9349477

[12] Qi X Li J Caussy C Teng GJ Loomba R. Epidemiology, screening, and co-management of type 2 diabetes mellitus and metabolic dysfunction-associated steatotic liver disease. *Hepatology*. (2026) 83:661–78. 10.1097/HEP.0000000000000913 38722246 PMC12904236

[13] Hagström H Shang Y Hegmar H Nasr P. Natural history and progression of metabolic dysfunction-associated steatotic liver disease. *Lancet Gastroenterol Hepatol*. (2024) 9:944–56. 10.1016/S2468-1253(24)00193-6 39243773

[14] Anstee QM Castera L Loomba R. Impact of non-invasive biomarkers on hepatology practice: past, present and future. *J Hepatol*. (2022) 76:1362–78. 10.1016/j.jhep.2022.03.026 35589256

[15] Eslam M Fan JG Yu ML Wong VW Cua IH Liu CJ et al. The Asian Pacific association for the study of the liver clinical practice guidelines for the diagnosis and management of metabolic dysfunction-associated fatty liver disease. *Hepatol Int*. (2025) 19:261–301. 10.1007/s12072-024-10774-3 40016576

[16] Lim TS Kim JK. Is liver biopsy still useful in the era of non-invasive tests? *Clin Mol Hepatol*. (2020) 26:302–4. 10.3350/cmh.2020.0081 32646204 PMC7364357

[17] Rinella ME Neuschwander-Tetri BA Siddiqui MS Abdelmalek MF Caldwell S Barb D et al. AASLD Practice Guidance on the clinical assessment and management of nonalcoholic fatty liver disease. *Hepatology*. (2023) 77:1797–835. 10.1097/HEP.0000000000000323 36727674 PMC10735173

[18] Hernaez R Lazo M Bonekamp S Kamel I Brancati FL Guallar E et al. Diagnostic accuracy and reliability of ultrasonography for the detection of fatty liver: a meta-analysis. *Hepatology*. (2011) 54:1082–90. 10.1002/hep.24452 21618575 PMC4197002

[19] Younossi ZM Zelber-Sagi S Lazarus JV Wong VW Yilmaz Y Duseja A et al. Global consensus recommendations for metabolic dysfunction-associated steatotic liver disease and steatohepatitis. *Gastroenterology*. (2025) 169:1017–32.e2. 10.1053/j.gastro.2025.02.044 40222485

[20] Stefan N Yki-Järvinen H Neuschwander-Tetri BA. Metabolic dysfunction-associated steatotic liver disease: heterogeneous pathomechanisms and effectiveness of metabolism-based treatment. *Lancet Diabetes Endocrinol*. (2025) 13:134–48. 10.1016/S2213-8587(24)00318-8 39681121

[21] Azzu V Vacca M Virtue S Allison M Vidal-Puig A. Adipose tissue-liver cross talk in the control of whole-body metabolism: implications in nonalcoholic fatty liver disease. *Gastroenterology*. (2020) 158:1899–912. 10.1053/j.gastro.2019.12.054 32061598

[22] Chen Z Yu R Xiong Y Du F Zhu S. A vicious circle between insulin resistance and inflammation in nonalcoholic fatty liver disease. *Lipids Health Dis*. (2017) 16:203. 10.1186/s12944-017-0572-9 29037210 PMC5644081

[23] Ahmed B Sultana R Greene MW. Adipose tissue and insulin resistance in obese. *Biomed Pharmacother*. (2021) 137:111315. 10.1016/j.biopha.2021.111315 33561645

[24] Zhang M Zhao E Sun G. Association between waist circumference and fatty liver disease in older adult population: a cross-sectional study in Urumqi. *Front Public Health*. (2025) 13:1620261. 10.3389/fpubh.2025.1620261 40697841 PMC12279773

[25] Janakiram N Puri S Thirumalai A. Waist circumference as a predictor of hepatic steatosis and fibrosis in the NHANES 2017-2018 cohort. *Metab Syndr Relat Disord*. (2026) 24:112–20. 10.1177/15578518261419363 41645541

[26] Cai J Lin C Lai S Liu Y Liang M Qin Y et al. Waist-to-height ratio, an optimal anthropometric indicator for metabolic dysfunction associated fatty liver disease in the Western Chinese male population. *Lipids Health Dis*. (2021) 20:145. 10.1186/s12944-021-01568-9 34706716 PMC8549212

[27] Wu X Mo J Yu J Zhang S Zheng D Chen X et al. The relationship between triglyceride-glucose index, triglyceride-glucose-body mass index, and the severity of hepatic steatosis and liver fibrosis in patients with MASLD: a cross-sectional study. *Front Nutr*. (2026) 13:1740308. 10.3389/fnut.2026.1740308 41835374 PMC12979549

[28] Chen Q Hu P Hou X Sun Y Jiao M Peng L et al. Association between triglyceride-glucose related indices and mortality among individuals with non-alcoholic fatty liver disease or metabolic dysfunction-associated steatotic liver disease. *Cardiovasc Diabetol*. (2024) 23:232. 10.1186/s12933-024-02343-7 38965572 PMC11225330

[29] Song S Zhang Y Qiao X Duo Y Xu J Peng Z et al. HOMA-IR as a risk factor of gestational diabetes mellitus and a novel simple surrogate index in early pregnancy. *Int J Gynaecol Obstet*. (2022) 157:694–701. 10.1002/ijgo.13905 34449903

[30] Xu Z Li C Wang N Song G. Association between the new TyG indicator-TyHGB and gestational diabetes mellitus: results from the case-control and prospective cohort studies. *J Transl Med*. (2025) 23:190. 10.1186/s12967-025-06166-2 39956901 PMC11831843

[31] Yang J Xie B Deng Z Zhang Z Zhou H. The triglyceride-high-density lipoprotein-glucose-body index: a superior novel biomarker for diabetic kidney disease in type 2 diabetes. *Front Endocrinol (Lausanne)*. (2026) 16:1749826. 10.3389/fendo.2025.1749826 41601932 PMC12832339

[32] Sun M Lin W Gong R Guo R Zhang M Yin Y et al. TyHGB combined with diagonal earlobe crease and traditional risk factors for constructing a diagnostic model of coronary heart disease. *Lipids Health Dis*. (2026) 25:66. 10.1186/s12944-026-02880-y 41606747 PMC12924619

[33] Lu A Xu Q. Association between the triglyceride-high-density lipoprotein-glucose-body index and new-onset stroke risk: a prospective cohort study. *Lipids Health Dis*. (2026) 25:143. 10.1186/s12944-026-02953-y 42002766 PMC13224718

[34] Zhao E Wang R Chen Y Xie H Gao Y Dong B et al. Evaluation of a new TyG indicator, TyHGB, in predicting non-alcoholic fatty liver disease: evidence from two independent populations. *Lipids Health Dis*. (2025) 24:342. 10.1186/s12944-025-02742-z 41131568 PMC12551267

[35] Umemura S Arima H Arima S Asayama K Dohi Y Hirooka Y et al. The Japanese society of hypertension guidelines for the management of hypertension (JSH 2019). *Hypertens Res*. (2019) 42:1235–481. 10.1038/s41440-019-0284-9 31375757

[36] Xie H Yan C Zheng Y Wu H. Assessment of ten insulin resistance surrogate indexes predicts new-onset cardiovascular disease incidence in patients with prediabetes or diabetes: insights from CHARLS data with machine learning analysis. *Glob Heart*. (2026) 21:17. 10.5334/gh.1532 41836049 PMC12985947

[37] Zhang Q Xu J Qian Y. Association between TyHGB and hyperuricemia and gout in patients with type-2 diabetes mellitus. *BMC Endocr Disord*. (2026) 26:178. 10.1186/s12902-026-02294-y 42062982 PMC13274143

[38] Han X Kong J Zhang H Zhao Y Zheng Y Wei C. Triglycerides mediate the influence of body mass index on non-alcoholic fatty liver disease in a non-obese chinese population with normal low-density lipoprotein cholesterol levels. *Obes Facts*. (2024) 17:191–200. 10.1159/000536447 38266508 PMC10987190

[39] Chung GE Oh S Ahn DW Kim SH Jung YJ Kim JW et al. Effects of additive interactions among obesity, visceral adiposity, and sarcopenia on nonalcoholic fatty liver disease. *Sci Rep*. (2023) 13:3628. 10.1038/s41598-023-30833-3 36869158 PMC9984466

[40] Unamuno X Gómez-Ambrosi J Rodríguez A Becerril S Frühbeck G Catalán V. Adipokine dysregulation and adipose tissue inflammation in human obesity. *Eur J Clin Invest*. (2018) 48:e12997. 10.1111/eci.12997 29995306

[41] Jung UJ Choi MS. Obesity and its metabolic complications: the role of adipokines and the relationship between obesity, inflammation, insulin resistance, dyslipidemia and nonalcoholic fatty liver disease. *Int J Mol Sci*. (2014) 15:6184–223. 10.3390/ijms15046184 24733068 PMC4013623

[42] Mikami K Endo T Sawada N Igarashi G Kimura M Hasegawa T et al. Leptin/adiponectin ratio correlates with hepatic steatosis but not arterial stiffness in nonalcoholic fatty liver disease in Japanese population. *Cytokine*. (2020) 126:154927. 10.1016/j.cyto.2019.154927 31756645

[43] Gugliucci A. Biomarkers of dysfunctional visceral fat. *Adv Clin Chem*. (2022) 109:1–30. 10.1016/bs.acc.2022.03.001 35953124

[44] Cossins BC van den Munckhof I Rutten JHW van der Graaf M Stienstra R Joosten LAB et al. Sex-specific association between adipose tissue inflammation and vascular and metabolic complications of obesity. *J Clin Endocrinol Metab*. (2023) 108:2537–49. 10.1210/clinem/dgad193 37014796 PMC10505527

[45] Arab JP Arrese M Trauner M. Recent insights into the pathogenesis of nonalcoholic fatty liver disease. *Annu Rev Pathol*. (2018) 13:321–50. 10.1146/annurev-pathol-020117-043617 29414249

[46] Sanders FW Griffin JL. De novo lipogenesis in the liver in health and disease: more than just a shunting yard for glucose. *Biol Rev Camb Philos Soc*. (2016) 91:452–68. 10.1111/brv.12178 25740151 PMC4832395

[47] Israelsen M Francque S Tsochatzis EA Krag A. Steatotic liver disease. *Lancet*. (2024) 404:1761–78. 10.1016/S0140-6736(24)01811-7 39488409

[48] Buzzetti E Pinzani M Tsochatzis EA. The multiple-hit pathogenesis of non-alcoholic fatty liver disease (NAFLD). *Metabolism*. (2016) 65:1038–48. 10.1016/j.metabol.2015.12.012 26823198

[49] Hsu CL Schnabl B. The gut-liver axis and gut microbiota in health and liver disease. *Nat Rev Microbiol*. (2023) 21:719–33. 10.1038/s41579-023-00904-3 37316582 PMC10794111

[50] Tilg H Adolph TE Moschen AR. Multiple parallel hits hypothesis in nonalcoholic fatty liver disease: revisited after a decade. *Hepatology*. (2021) 73:833–42. 10.1002/hep.31518 32780879 PMC7898624

