# Supplementary material for: Waist circumference partially mediates the non-linear association between TyHGB and MASLD in non-diabetic Japanese adults
Source: Front Nutr. 2026 Jul 24;13:1915937. doi: 10.3389/fnut.2026.1915937 (PMC13447069; doi:10.3389/fnut.2026.1915937)
Supplement: Supplementary file 1 [file Table_1.docx]

Supplementary Material

**Supplementary Tables**

**Supplemental Table 1.** The best threshold, sensitivities, specificities, and AUC of each parameter for screening MASLD in the general population and subgroup analysis for sex, age, BMI, TG, hypertension, and WC.

|  | **AUC** | **95% CI** | **Best threshold** | **Specificity** | **Sensitivity** |
| --- | --- | --- | --- | --- | --- |
| **General population** | | | | | |
| TyHGB | 0.8882 | 0.8819-0.8945 | 7.5974 | 0.7661 | 0.8577 |
| TyG | 0.8279* | 0.8188-0.8370 | 8.2064 | 0.7198 | 0.7844 |
| AIP | 0.8369* | 0.8282-0.8456 | 0.1930 | 0.7339 | 0.7873 |
| **Subgroup analysis stratified by sex** | | | | | |
| **Men** | | | | | |
| TyHGB | 0.9155 | 0.9046-0.9264 | 7.1131 | 0.8160 | 0.8740 |
| TyG | 0.8319* | 0.8121-0.8517 | 7.9948 | 0.7225 | 0.7969 |
| AIP | 0.8332* | 0.8131-0.8532 | 0.0549 | 0.7435 | 0.7892 |
| **Women** | | | | | |
| TyHGB | 0.8272 | 0.8168-0.8375 | 8.0235 | 0.7071 | 0.8027 |
| TyG | 0.7674* | 0.7547-0.7801 | 8.4420 | 0.7268 | 0.6832 |
| AIP | 0.7731* | 0.7607-0.7856 | 0.3112 | 0.7077 | 0.7097 |
| **Subgroup analysis stratified by age (years)** | | | | | |
| **< 40** | | | | | |
| TyHGB | 0.9157 | 0.9067-0.9247 | 7.4095 | 0.7868 | 0.9021 |
| TyG | 0.8609* | 0.8466-0.8752 | 8.1722 | 0.7793 | 0.7951 |
| AIP | 0.8682* | 0.8548-0.8816 | 0.1642 | 0.7635 | 0.8196 |
| **≥ 40** | | | | | |
| TyHGB | 0.8707 | 0.8622-0.8792 | 7.6017 | 0.7334 | 0.8562 |
| TyG | 0.8061* | 0.7943-0.8180 | 8.2064 | 0.6713 | 0.7913 |
| AIP | 0.8171* | 0.8057-0.8285 | 0.2322 | 0.7403 | 0.7481 |
| **Subgroup analysis stratified by BMI (kg/m^2^)** | | | | | |
| **< 25** | | | | | |
| TyHGB | 0.8809 | 0.8717-0.8901 | 7.2183 | 0.7186 | 0.8920 |
| TyG | 0.8311* | 0.8179-0.8443 | 8.1443 | 0.7086 | 0.8060 |
| AIP | 0.8391* | 0.8265-0.8518 | 0.1470 | 0.7121 | 0.8130 |
| **≥ 25** | | | | | |
| TyHGB | 0.7222 | 0.7006-0.7438 | 8.3179 | 0.5850 | 0.7569 |
| TyG | 0.6944* | 0.6721-0.7167 | 8.5758 | 0.7367 | 0.5604 |
| AIP | 0.6963* | 0.6741-0.7185 | 0.3401 | 0.6544 | 0.6419 |
| **Subgroup analysis stratified by TG (mmol/L)** | | | | | |
| **< 1.7** | | | | | |
| TyHGB | 0.8817 | 0.8743-0.8891 | 7.3775 | 0.7520 | 0.8620 |
| TyG | 0.7954* | 0.7841-0.8067 | 8.1122 | 0.6960 | 0.7564 |
| AIP | 0.8085* | 0.7977-0.8193 | 0.1413 | 0.7139 | 0.7660 |
| **≥ 1.7** |  |  |  |  |  |
| TyHGB | 0.6642 | 0.6324-0.6959 | 10.4700 | 0.4923 | 0.7962 |
| TyG | 0.6151* | 0.5826-0.6476 | 9.0352 | 0.4521 | 0.7461 |
| AIP | 0.6162* | 0.5835-0.6488 | 0.6420 | 0.4904 | 0.7085 |
| **Subgroup analysis stratified by Hypertension** | | | | | |
| **No** |  |  |  |  |  |
| TyHGB | 0.8879 | 0.8812-0.8946 | 7.3775 | 0.7277 | 0.8956 |
| TyG | 0.8271* | 0.8173-0.8368 | 8.2064 | 0.7281 | 0.7754 |
| AIP | 0.8363* | 0.8269-0.8457 | 0.1475 | 0.6946 | 0.8259 |
| **Yes** |  |  |  |  |  |
| TyHGB | 0.8179 | 0.7881-0.8477 | 8.3135 | 0.7306 | 0.7716 |
| TyG | 0.7503* | 0.7151-0.7855 | 8.4358 | 0.7063 | 0.7253 |
| AIP | 0.7669* | 0.7331-0.8007 | 0.2481 | 0.6359 | 0.7809 |
| **Subgroup analysis stratified by WC** | | | | | |
| **Normal** |  |  |  |  |  |
| TyHGB | 0.8920 | 0.8847-0.8993 | 7.3781 | 0.7403 | 0.8910 |
| TyG | 0.8396* | 0.8289-0.8504 | 8.2064 | 0.7323 | 0.7876 |
| AIP | 0.8515* | 0.8414-0.8616 | 0.1470 | 0.6986 | 0.8462 |
| **Elevated** |  |  |  |  |  |
| TyHGB | 0.8025 | 0.7824-0.8225 | 8.0686 | 0.6825 | 0.7914 |
| TyG | 0.7518* | 0.7296-0.8225 | 8.3140 | 0.6582 | 0.7121 |
| AIP | 0.7567* | 0.7347-0.7787 | 0.3207 | 0.7587 | 0.6212 |

* *P* < 0.01, compared with TyHGB. **Abbreviations:** MASLD, Metabolic dysfunction associated steatotic liver disease; TyHGB, Triglyceride high-density cholesterol-glucose body index; BMI, Body mass index; WC, Waist circumference; AIP, Atherogenic index of plasma; TG, Triglycerides; TyG, Triglyceride-glucose; CI, Confidence interval; ROC, Receiver operating characteristic; AUC, Area under the curve.

**Supplemental Table 2.** Analysis of the mediation by WC of the associations of TyHGB with the risk of MASLD

|  | **Mediation effect (95% CI), *P* value** | | |  |
| --- | --- | --- | --- | --- |
|  | Total effect | Indirect effect | Direct effect | Mediation |
| **WC** | 0.062（0.056,0.071),  0.002 | 0.029 (0.026,0.033),  < 0.001 | 0.033 (0.028,0.040),  0.002 | 46.40%,  0.002 |
| **Subgroup analysis stratified by sex** | | | | |
| **Men** | 0.025 (0.006,0.027),  0.004 | 0.012 (0.003,0.014),  0.006 | 0.012 (0.003,0.015),  0.004 | 49.41%,  0.004 |
| **Women** | 0.101 (0.087,0.120),  < 0.001 | 0.043 (0.037,0.049),  < 0.001 | 0.058 (0.047,0.074),  < 0.001 | 42.39%,  < 0.001 |
| **Subgroup analysis stratified by age (years)** | | | | |
| **< 40** | 0.053 (0.038,0.068),  0.002 | 0.025(0.019,0.032),  0.002 | 0.028 (0.017,0.039),  0.004 | 46.67%,  < 0.001 |
| **≥ 40** | 0.067 (0.056,0.079),  < 0.001 | 0.031 (0.027,0.035),  < 0.001 | 0.036 (0.028,0.046),  < 0.001 | 46.23%,  < 0.001 |
| **Subgroup analysis stratified by BMI (kg/m^2^)** | | | | |
| **< 25** | 0.027 (0.022,0.030),  < 0.001 | 0.011(0.009,0.013),  0.002 | 0.015 (0.012,0.018),  0.002 | 42.67%,  < 0.001 |
| **≥ 25** | 0.122 (0.093,0.155),  < 0.001 | 0.010 (0.005,0.014),  < 0.001 | 0.112 (0.083,0.147),  < 0.001 | 7.83%,  < 0.001 |
| **Subgroup analysis stratified by TG (mmol/L)** | | | | |
| **< 1.7** | 0.086 (0.080,0.094),  0.004 | 0.041 (0.036,0.046),  < 0.001 | 0.045 (0.039,0.051),  0.016 | 47.42%,  0.004 |
| **≥ 1.7** | 0.050 (0.026,0.080),  < 0.001 | 0.018(0.010,0.028),  < 0.001 | 0.032 (0.011,0.058),  0.006 | 35.88%,  < 0.001 |
| **Subgroup analysis stratified by Hypertension** | | | | |
| **No** | 0.057 (0.050,0.065),  < 0.001 | 0.028 (0.025,0.031),  < 0.001 | 0.030 (0.024,0.036),  < 0.001 | 48.39%,  < 0.001 |
| **Yes** | 0.151 (0.106,0.201),  < 0.001 | 0.043 (0.025,0.064),  < 0.001 | 0.109 (0.063,0.161),  < 0.001 | 28.13%,  < 0.001 |
| **Subgroup analysis stratified by WC** | | | | |
| **Normal** | 0.038 (0.032,0.043),  < 0.001 | 0.016 (0.014,0.018),  < 0.001 | 0.022 (0.017,0.026),  < 0.001 | 42.31%,  < 0.001 |
| **Elevated** | 0.127 (0.092,0.179),  < 0.001 | 0.014 (0.007,0.022),  < 0.001 | 0.114 (0.077,0.165),  < 0.001 | 10.84%,  < 0.001 |

Age, sex, hypertension, smoking status, drinking status, exercise status, HbA1c, ALT, AST, GGT, and LDL-C were adjusted. In the subgroup analysis for sex, the model was not adjusted for sex. In the subgroup analysis for age, the model was not adjusted for age. In the subgroup analysis for hypertension, the model was not adjusted for hypertension. Elevated WC was defined as ≥ 90 cm in men and ≥ 80 cm in women; normal WC was defined as < 90 cm in men and < 80 cm in women.

**Abbreviations:** MASLD, Metabolic dysfunction associated steatotic liver disease; TyHGB, Triglyceride high-density cholesterol-glucose body index; BMI, Body mass index; WC, Waist circumference; HbA1c, Glycosylated hemoglobin; ALT, Alanine aminotransferase; AST, Aspartate aminotransferase; GGT, Gamma-glutamyl transpeptidase; TG, Triglycerides; LDL-C, Low-density lipoprotein cholesterol; CI, Confidence interval.

**Supplemental Table 3.** Sensitivity analysis of the mediation effect of WC on the association between a modified TyHGB index (TyHGB_noBMI, excluding the BMI component) and MASLD risk in the general population.

|  | **Mediation effect (95% CI), *P* value** | | |  |
| --- | --- | --- | --- | --- |
|  | Total effect | Indirect effect | Direct effect | Mediation |
| **WC** | 0.055（0.049,0.063),  < 0.001 | 0.022 (0.020,0.025),  < 0.001 | 0.033 (0.028,0.039),  < 0.001 | 40.44%,  < 0.001 |

Age, sex, hypertension, smoking status, drinking status, exercise status, HbA1c, ALT, AST, GGT, and LDL-C were adjusted.

**Abbreviations:** MASLD, Metabolic dysfunction associated steatotic liver disease; TyHGB, Triglyceride high-density cholesterol-glucose body index; BMI, Body mass index; WC, Waist circumference; HbA1c, Glycosylated hemoglobin; ALT, Alanine aminotransferase; AST, Aspartate aminotransferase; GGT, Gamma-glutamyl transpeptidase; TG, Triglycerides; LDL-C, Low-density lipoprotein cholesterol; CI, Confidence interval.

**Supplemental Table 4. Distribution of participants and MASLD cases stratified by global TyHGB inflection point**

| **TyHGB strata** | **Total participants** | **Number of MASLD cases** | **MASLD prevalence** |
| --- | --- | --- | --- |
| **< 9.70** | **12397** | **1318** | **10.63%** |
| **≥ 9.7** | **1285** | **769** | **59.75%** |

**Note: The inflection point was identified at 9.70 using two-piecewise linear regression.**

**Abbreviations:** MASLD, Metabolic dysfunction associated steatotic liver disease; TyHGB, Triglyceride high-density cholesterol-glucose body index
